# A scoping review of the unassisted physical exam conducted over synchronous audio-video telemedicine

**DOI:** 10.1186/s13643-022-02085-1

**Published:** 2022-10-13

**Authors:** Peter Yao, Mahad Adam, Sunday Clark, Hanson Hsu, Michael Stern, Rahul Sharma, Keith Mages, Peter Greenwald, Neel Naik

**Affiliations:** 1 Weill Cornell Medical College, New York, NY USA; 2 Columbia University, New York, NY USA; 3 Boston University School of Medicine, Boston, MA USA; 4 Department of Emergency Medicine, Weill Cornell Medicine, 525 E 68th Street, New York, NY 10065 USA; 5 Samuel J. Wood Library and C.V. Starr Biomedical Information Center, Weill Cornell Medicine, New York, NY USA

**Keywords:** Telemedicine, Physical examination, Telehealth, Scoping review

## Abstract

**Background:**

This scoping review aims to provide a broad overview of the research on the unassisted virtual physical exam performed over synchronous audio-video telemedicine to identify gaps in knowledge and guide future research.

**Methods:**

Searches for studies on the unassisted virtual physical exam were conducted in 3 databases. We included primary research studies in English on the virtual physical exam conducted via patient-to-provider synchronous, audio-video telemedicine in the absence of assistive technology or personnel. Screening and data extraction were performed by 2 independent reviewers.

**Results:**

Seventy-four studies met inclusion criteria. The most common components of the physical exam performed over telemedicine were neurologic (38/74, 51%), musculoskeletal (10/74, 14%), multi-system (6/74, 8%), neuropsychologic (5/74, 7%), and skin (5/74, 7%). The majority of the literature focuses on the telemedicine physical exam in the adult population, with only 5% of studies conducted specifically in a pediatric population. During the telemedicine exam, the patients were most commonly located in outpatient offices (28/74, 38%) and homes and other non-clinical settings (25/74, 34%). Both patients and providers in the included studies most frequently used computers for the telemedicine encounter.

**Conclusions:**

Research evaluating the unassisted virtual physical exam is at an early stage of maturity and is skewed toward the neurologic, musculoskeletal, neuropsychologic, and skin exam components. Future research should focus on expanding the range of telemedicine exam maneuvers studied and evaluating the exam in the most relevant settings, which for telemedicine is trending toward exams conducted through mobile devices and in patients’ homes.

**Supplementary Information:**

The online version contains supplementary material available at 10.1186/s13643-022-02085-1.

## Background

Although telemedicine has existed for decades, the pandemic inspired a new need to avoid face-to-face contact in both providers and patients. This, coupled with changes in payment and regulatory frameworks designed to encourage the use of telemedicine, resulted in a meteoric increase in the use of real-time video interactions for medical care [1, 2]. It appears that many of these regulatory changes may be long-lasting. The US Centers for Medicare and Medicaid Services has extended the payment changes through the end of 2023. Even if some of the payment and regulatory alterations put in place to facilitate telemedicine revert, it seems unlikely that the environment will return to the way it was before the pandemic. Many are convinced that the public, now familiar with the convenience of telemedicine, will continue to choose providers that offer telemedicine for at least a portion of their care.

Although many observers view the expansion of telemedicine capability favorably, the rapid substitution of telemedicine for face-to-face encounters has led to concerns regarding quality, access equity, and safety [3]. Academic discussion about what constitutes (and how to measure) high-quality video telemedicine has only just begun [4–6].

One criticism leveled at telemedicine is that a video encounter precludes the performance of a physical exam. The physical exam is one of the core pillars of information gathering in medicine; critics correctly assert, however, that it is a mistake to assume that video interaction precludes a physical exam [7]. Experience with telemedicine has shown that it is indeed possible to perform a physical examination over video, albeit a different sort of exam than that which occurs in person. National organizations have even begun to teach how to do an examination over video at the graduate medical education level [8]. Since it is a relatively new technique, the specific strengths and limitations of the video exam have not been explored. Hence, it is appropriate to investigate differences in examination over video as compared to face-to-face medical evaluation.

Like the face-to-face encounter, telemedicine examinations may use tools or technology to facilitate the exam [9], but even in the absence of devices such as web-enabled stethoscopes and otoscopes, it is possible to perform examinations and obtain actionable information. Visual inspection alone can yield crucial data, and many elements of a traditional exam that are identified with the examiner’s hands appear to be well approximated by providers instructing patients to assist them with the exam. For example, a tele-pediatrician can ask their young patient to jump up and down when evaluating them for appendicitis. Although providers of the real-time video evaluation are developing practice patterns and sharing their experiences about the patient exam, there has been no systematic assessment of what is and is not known on this topic.

We sought to summarize the state of the published literature related to physical exam when conducted over live video. To this end, we conducted a scoping review to identify, appraise, and synthesize relevant data on the physical exam conducted via patient-to-provider synchronous, audio-video telemedicine in the absence of assistive technology or trained medical personnel on the patient side.

## Methods

The scoping review methods are reported using the Preferred Reporting Items for Systematic reviews and Meta-Analyses extension for Scoping Reviews (PRISMA-ScR) Checklist (see Additional file 1: PRISMA-ScR Checklist) [10].

### Search strategy

A comprehensive literature search on the telemedicine physical exam was collaboratively developed by a research team member [PY] and medical librarian [KM], in consultation with senior investigators [PG, NN]. The initial search was performed on March 17, 2020, via OVID MEDLINE® ALL. It was then translated and rerun on OVID EMBASE and The Cochrane Library (Cochrane Database of Systematic Reviews, Cochrane Central Register of Controlled Trials (CENTRAL), Cochrane Methodology Register). Search terms in each database included all MeSH terms and/or keywords associated with our research question, clustered around the key areas: (1) telemedicine and (2) physical examination. Search terms were joined using Boolean operators “OR” and “AND,” as appropriate. Search dates were limited to articles published after 1990 for relevance to current telemedicine practices. The full Ovid MEDLINE search strategy is available in Supplementary Appendix 1.

### Study selection

Covidence (Veritas Health Innovation, Melbourne, Australia), a systematic review management software, was used for data management. After excluding duplicates using the automated Covidence workflow, reviewers [PY, HH, NN, MA, PG, MS] independently screened the titles and abstracts of the retrieved. Each citation was reviewed by two members of the research team, with consensus needed to move the citation forward to the next phase of screening. Discrepancies were resolved through group consensus. Inclusion and exclusion criteria were determined a priori. We included English primary research studies in which: (1) patients received any subset of a telemedicine physical exam by a licensed medical provider, (2) the exam was conducted over real-time audiovisual telemedicine, and (3) the exam was described in detail or validated by appropriate comparison to an in-person exam or another reference standard. We excluded studies in which (1) the physical exam was performed with the assistance of another medical provider or tele-presenter on the patient side and (2) the exam was assisted by equipment or technology obtained specifically for the purpose of the virtual physical exam. Excluded assistive equipment included but was not limited to equipment provided by the study to the patient for use in the virtual exam or equipment purchased by the patient specifically for the virtual exam. For example, a retinal camera or a dermatoscope provided to the patient for the purpose of the virtual exam would be excluded. Common medical devices sometimes found in the home, such as blood pressure cuffs, thermometers, glucometers, peak flow devices, and pulse oximeters, available to the patient during the virtual exam but not specifically intended for use only in the virtual exam, were not excluded.

Following the initial title and abstract screening phase, reviewers [PY, HH, NN, MA, PG, MS] independently screened the full text of citations using the same process outlined above.

### Data extraction

A data extraction sheet was developed and pilot-tested iteratively to ensure relevant and consistent information capture. Definitions for each data extraction field were agreed upon by all authors (Supplementary Appendix 2). The following information was collected: study and patient characteristics, telemedicine encounter setting, physical exam component studied, diagnostic context, and validation measure of physical exam component if such a measure was reported. Data extraction was performed on included articles using Covidence and then exported for analysis in Microsoft Excel. Data extraction was performed by a team member [PY], and a second team member [HH, NN, MA, PG, MS, or SC] independently verified the extracted data.

## Results

**Fig. 1:**
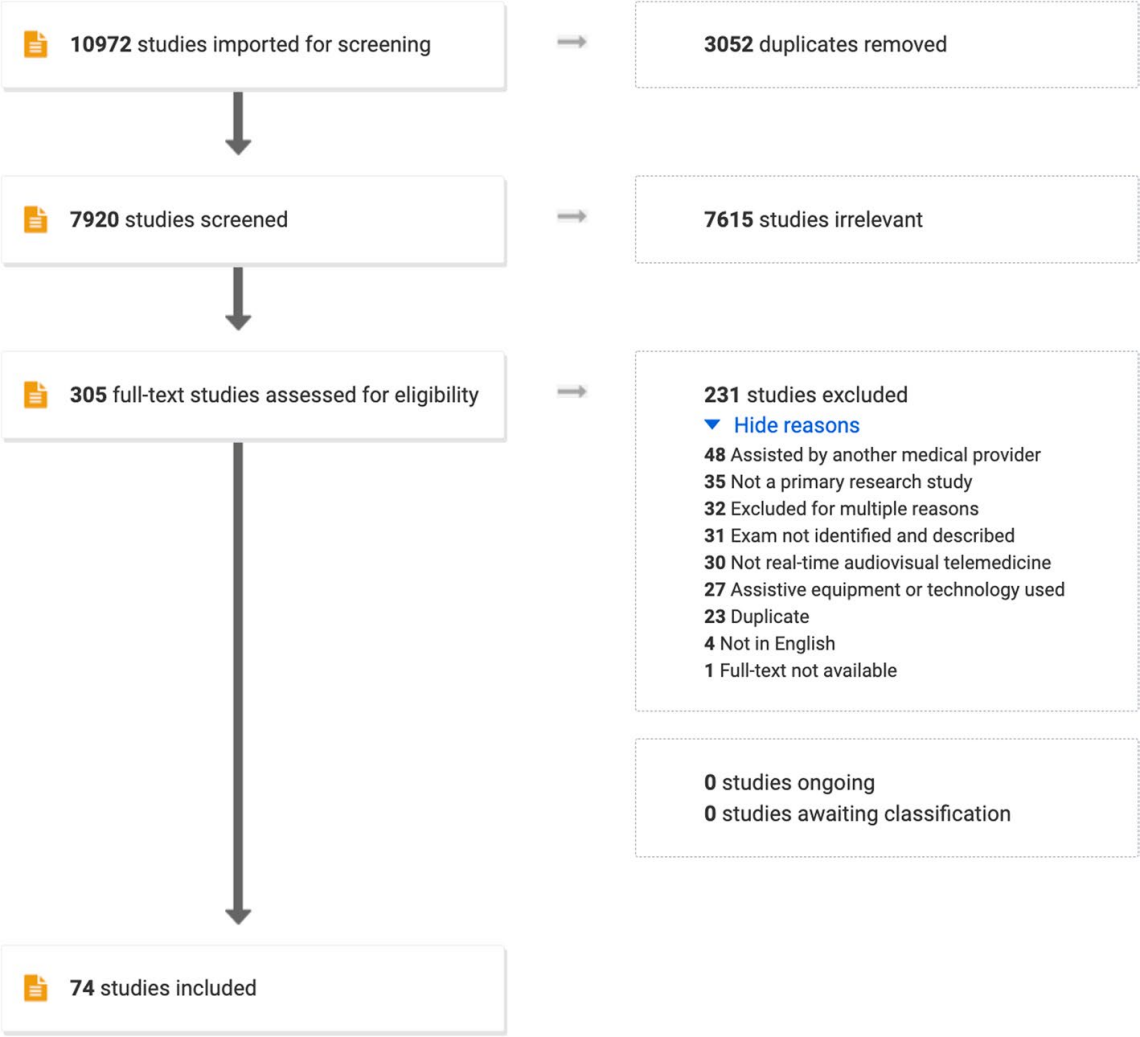
Preferred reporting items for systematic reviews and meta-analyses (PRISMA) flow diagram

The literature search yielded 7920 unique results after de-duplication. Following the title and abstract screen, 7615 studies were excluded. Three hundred five studies underwent full-text review. Seventy-four studies ultimately met all inclusion criteria. Citations for all included studies are available in Supplementary Appendix 3. The PRISMA flow diagram outlining the study selection process is available in Fig. 1. A summary of the included studies with details on virtual exam maneuvers and study results is available in Table 1.

**Table 1:** Summary of physical exam maneuvers and outcomes of the included studies organized by physical exam category. In the “Equivalence” column, “equivalent” means the telemedicine exam and the in-person exam were equivalent, “inferior” means the telemedicine exam was inferior to the in-person exam, and “NS” means the equivalence between the telemedicine exam and the in-person exam was not specified

Category of physical exam	Study	Title	Specific exam maneuvers	Outcome measure	Equivalence
Abdomen	Nachum 2019	Use of Physician-Guided Patient Self-Examination to Diagnose Appendicitis: A Telemedicine Case Report.	Abdominal self-palpation	Comparison against in-person exam	Equivalent
Cardiovascular	Cox 2013	Assessing exercise capacity using telehealth: A feasibility study in adults with cystic fibrosis	3-min step test	Comparison against in-person exam	Equivalent
Cardiovascular	Seuren 2020	Physical Examinations via Video for Patients With Heart Failure: Qualitative Study Using Conversation Analysis	Fluid retention (by the patient or relative feeling for leg edema), blood pressure with pulse rate and rhythm (using a self-inflating blood pressure monitor incorporating an irregular heartbeat indicator and put on by the patient or relative), oxygen saturation (using a finger clip device)	Explore the opportunities and challenges of remote physical examination of patients with heart failure using video-mediated communication technology	NS
Head and neck	Akhtar 2018	Telemedicine Physical Examination Utilizing a Consumer Device Demonstrates Poor Concordance with In-Person Physical Examination in Emergency Department Patients with Sore Throat: A Prospective Blinded Study	Oropharyngeal coloration, posterior pharynx asymmetry, uvular deviation, oropharynx exudate, swelling, and ulceration, head and neck lymph nodes	Comparison against in-person exam	Inferior
Multi-system	Bell 2016	Telemedicine versus face-to-face evaluations by respiratory therapists of mechanically ventilated neonates and children: A pilot study	Pressure control, PEEP, mean airway pressure, breathing frequency, FIO2, inspiratory to expiratory time ratio (I-E ratio), tidal volume (VT), minute ventilation, oxygen saturation, presence of patient-triggered breaths, the need for suctioning or increased ventilator support	Comparison against in-person exam	Equivalent
Multi-system	Duffy 1997	Telemedicine and the diagnosis of speech and language disorders	Oral mechanism exam, motor speech exam, language exam	Summarize our speech pathology consultation-by-satellite experience, with emphasis on the methods used, information obtained, difficulties encountered and overcome, and types of speech and language problems that have been evaluated through this medium	NS
Multi-system	Gallar 2007	Two-year experience with telemedicine in the follow-up of patients in home peritoneal dialysis	Catheter exit site appearance, dialysis fluid appearance, leg edema	Utility and cost-effectiveness of telemedicine in peritoneal dialysis	NS
Multi-system	Jenkins 2001	Assessing elderly patients with congestive heart failure via in-home interactive telecommunication	Assessment of respiratory effort, retraction, color of lips and nail beds, pedal and ankle edema	Comparison against in-person exam	Equivalent
Multi-system	Schoenfeld 2016	Variation in quality of urgent health care provided during commercial virtual visits	Ability to walk, tenderness at lateral malleolus, fever, tonsillar exudates, tender cervical adenopathy, suprapublic/flank/back pain	Completeness of histories and physical examinations via telemedicine, the correct diagnosis (vs an incorrect or no diagnosis), and adherence to guidelines of key management decisions.	Inferior
Multi-system	Siew 2016	Reliability of telemedicine in the assessment of seriously ill children	Respiratory Observation Checklist consisting of age-appropriate tachypnea, perioral cyanosis, nasal flaring, tripoding, thoracoabdominal asynchrony, supraclavicular, substernal, and intercostal retractions, mental status, and overall impression of respiratory distress. Yale Observation Scale consisting of quality of cry, reaction to parent stimulation, state variation or ability to be aroused, color, hydration status, and response to social overtures.	Comparison against in-person exam	Equivalent
Musculoskeletal	Abel 2017	Can Telemedicine Be Used for Adolescent Postoperative Knee Arthroscopy Follow-up?	Knee range of motion, incision color, effusion size	Comparison against in-person exam	Equivalent
Musculoskeletal	Cabana 2010	Interrater agreement between telerehabilitation and face-to-face clinical outcome measurements for total knee arthroplasty	Scar assessment, swelling of knee joint, muscle strength, locomotion (Tinetti test), balance (Berg test)	Comparison against in-person exam	Equivalent
Musculoskeletal	Goldstein 2019	Video examination via the smartphone: A reliable tool for shoulder function assessment using the constant score.	Constant score (CS)—pain, activity level, arm positioning, strength (assisted by spring device, should exclude this), range of motion	Comparison against in-person exam	Equivalent
Musculoskeletal	Good 2012	Skype: A tool for functional assessment in orthopaedic research	Oxford shoulder score, Constant shoulder score	Comparison against in-person exam (the agreement had to do with the angle measurements)	Equivalent
Musculoskeletal	Jacobson 2016	Telehealth Videoconferencing for Children With Hemophilia and Their Families: A Clinical Project	Appearance of blood, swelling, range of motion	Evaluate the feasibility of using telehealth videoconferencing in children with severe hemophilia in the home setting.	NS
Musculoskeletal	Lade 2012	Validity and reliability of the assessment and diagnosis of musculoskeletal elbow disorders using telerehabilitation	Observations, self-palpate, conduct range of motion tests with self-applied overpressure, perform self-resisted static muscle tests, self-applied special orthopedic tests, self-applied ULNT tests	Comparison against in-person exam	Equivalent
Musculoskeletal	Richardson 2017	Physiotherapy assessment and diagnosis of musculoskeletal disorders of the knee via telerehabilitation	Self-palpation, self-applied modified orthopedic tests such as pain intensity with passive varus force by verbal guidance, active movements	Comparison against in-person exam	Equivalent
Musculoskeletal	Russell 2013	Internet-based physical assessment of people with Parkinson disease is accurate and reliable: a pilot study.	Timed stance test, Timed Up and Go test, step test, steps in 360 degree turn, Berg Balance Scale, lateral and functional reach test	Comparison against in-person exam	Equivalent
Musculoskeletal	Steele 2012	Assessment and diagnosis of musculoskeletal shoulder disorders over the internet	Postural analysis, joint palpation, range of motion (ROM) testing at the shoulder and adjacent joints, static muscle tests (SMTs), special orthopedic tests, neural testing, Hawkins-Kennedy impingement test	Comparison against in-person exam	Equivalent
Musculoskeletal	Turner 2018	Case Studies in Physical Therapy: Transitioning A "Hands-On" Approach into A Virtual Platform.	Range of motion	Feasibility of performing musculoskeletal exam over telemedicine	NS
Neurologic	Abdolahi 2016	A feasibility study of conducting the Montreal Cognitive Assessment remotely in individuals with movement disorders	MoCA	Comparison against in-person exam	Equivalent
Neurologic	Ali 2013	Accuracy of remote video cellphone evaluation of stroke deficits using california brief stroke scale	California Brief Stroke Scale (CABSS), a prehospital telemedicine instrument to rate stroke severity	Comparison against in-person exam	Equivalent
Neurologic	Andrzejewski 2015	Determining the reliability of performing the modified unified Parkinson's disease rating scale (UPDRS) remotely in a pilot virtual visit study in the home	Modified (excluding rigidity and postural instability) Unified Parkinson’s disease Rating Scale (mUPDRS)	Comparison against in-person exam	Equivalent
Neurologic	Ball 1993	Preliminary evaluation of a low-cost VideoConferencing (LCVC) system for remote cognitive testing of adult psychiatric patients	MMSE	Comparison against in-person exam	Equivalent
Neurologic	Bove 2019	Toward a low-cost, in-home, telemedicine-enabled assessment of disability in multiple sclerosis	Expanded Disability Status Scale (EDSS)	Comparison against in-person exam	Equivalent
Neurologic	Bull 2014	Conducting the montreal cognitive assessment remotely in Huntington's disease	MoCA	Comparison against in-person exam	Equivalent
Neurologic	Bull 2014	Monitoring fluctuations in motor function and mood hourly in Parkinson's disease via telemedicine	Modified version the Unified Parkinson’s disease Rating Scale (mUPDRS)	Correlation of mUPDRS with subjective mood and mobility ratings by patient on 1–10 scale	Equivalent
Neurologic	Carotenuto 2018	Cognitive Assessment of Patients With Alzheimer's Disease by Telemedicine: Pilot Study.	Italian versions of the MMSE and Alzheimer’s Disease Assessment Scale cognitive subscale (ADAS-cog)	Comparison against in-person exam	Equivalent
Neurologic	Castanho 2016	Assessing Cognitive Function in Older Adults Using a Videoconference Approach	The Telephone Interview for Cognitive Status-Modified--Portuguese version (TICSM-PT)	Comparison of videoconference exam against telephone and in-person exam	Equivalent
Neurologic	Cullum 2006	Feasibility of telecognitive assessment in dementia	MMSE, Hopkins Verbal Learning Test-Revised, Clock Drawing Test, Digit Span, Category Fluency (fruits and vegetables), letter fluency (FAS and CFL versions), 15-item versions of the Boston Naming Test	Comparison against in-person exam	Equivalent
Neurologic	Davis 2014	Teleneurology: Successful Delivery of Chronic Neurologic Care to 354 Patients Living Remotely in a Rural State	Full neurologic exam with the exception of deep tendon reflexes, a careful sensory exam, retinal exam, or complete oral exam of palate movement	Patient satisfaction with teleneurology care as measured by a performance improvement satisfaction questionnaire	NS
Neurologic	Davis 2019	Using Teleneurology to Deliver Chronic Neurologic Care to Rural Veterans: Analysis of the First 1,100 Patient Visits	Mental status exam, cranial nerve exam (eye movements, nystagmus, eye closure strength, speech, protrusion of their tongue, and neck rotation), motor exam (bradykinesia and involuntary movement assessment, patients were asked to hold their arms outstretched, perform repetitive movements with fingers and hands, stand up from their chair without using their arms, and walk around the room), coordination and balance (walk tandem, perform a Romberg test, rapidly move their fingers in sequence, and perform finger to nose and rapid alternating movement tests)	Survey measuring quality of care, ease of communication, satisfaction, and staff’s ability to deliver same quality care as in person.	NS
Neurologic	DeYoung 2019	The reliability of the Montreal Cognitive Assessment using telehealth in a rural setting with veterans.	Montreal Cognitive Assessment	Comparison against in-person exam	Equivalent
Neurologic	Dorsey 2015	Feasibility of virtual research visits in fox trial finder	Montreal Cognitive Assessment, MDS-UPDRS (excluding rigidity and balance)	Determine feasibility of virtual research visits, validate self-reported diagnosis of volunteers, and gauge satisfaction of neurologists and patients with visit through survey	NS
Neurologic	Fraint 2019	Reliability, feasibility and satisfaction of telemedicine evaluations for Cervical Dystonia.	Toronto Western Spasmodic Torticollis Rating Scale (TWSTRS) motor severity subscale	Comparison against in-person exam	Equivalent
Neurologic	Hubble 1993	Interactive video conferencing: a means of providing interim care to Parkinson's disease patients.	Unified PD Rating Scale (UPDRS), Hoehn and Yahr score	Comparison against in-person exam	Equivalent
Neurologic	Korn 2017	Virtual visits for Parkinson disease: A multicenter noncontrolled cohort	Parkinson’s disease-focused exam	The primary outcome measures were feasibility, as measured by the proportion of visits completed as scheduled, and the 6-month change in quality of life, as measured by the Parkinson’s Disease Questionnaire 39. Additional outcomes included satisfaction with visits and interest in future virtual visits.	NS
Neurologic	Lindauer 2017	Dementia Care Comes Home: Patient and Caregiver Assessment via Telemedicine	MoCA	Comparison against in-person exam	Equivalent
Neurologic	Loh 2007	Development of a telemedicine protocol for the diagnosis of Alzheimer's disease	Standardized Mini Mental State Examination, Geriatric Depression Scale, Katz assessment of Activities of Daily Living, Instrumental ADL assessment, the Informant Questionnaire for Cognitive Decline in the Elderly	comparison against in-person exam, agreement for diagnosis of Alzheimer's between telemedicine and in-person exam	Equivalent
Neurologic	McEachern 2008	Reliability of the MMSE administered in-person and by telehealth	Mini-mental status examination (MMSE)	Comparison against in-person exam	Equivalent
Neurologic	Montani 1997	Psychological impact of a remote psychometric consultation with hospitalized elderly people	Mini-mental state exam (MMSE), clock face test (CFT)	Comparison against in-person exam	Inferior
Neurologic	MunroCullum 2014	Teleneuropsychology: evidence for video teleconference-based neuropsychological assessment	Mini-Mental State Examination (MMSE), Hopkins Verbal Learning Test-Revised, Digit Span forward and backward, short form Boston Naming Test, Letter and Category Fluency, Clock Drawing	Comparison against in-person exam	Equivalent
Neurologic	Palsbo 2007	Televideo assessment using Functional Reach Test and European Stroke Scale	European Stroke Scale (ESS), the Functional Reach Test (FRT)	Comparison against in-person exam	Equivalent
Neurologic	Park 2017	Korean Version of the Mini-Mental State Examination Using Smartphone: A Validation Study	Korean version of the Mini-Mental State Examination (MMSE-K)	Comparison against in-person exam	Equivalent
Neurologic	Rudolph 2014	Tablet computers address limitations of telestroke systems	NIH stroke scale	Comparison against in-person exam	Equivalent
Neurologic	Shores 2004	Identifying undiagnosed dementia in residential care veterans: Comparing telemedicine to in-person clinical examination	Focused neurological exam (gait, eye movements, hand tremor, and frontal release signs), short Blessed, three world recall, clock drawing	Agreement between in-person and telemedicine exams on diagnosis of dementia	Equivalent
Neurologic	Stillerova 2016	Remotely assessing symptoms of Parkinson's disease using videoconferencing: A feasibility study	Movement Disorder Society Unified Parkinson’s Disease Rating Scale (MDS-UPDRS)	Comparison against in-person exam	Inferior
Neurologic	Stillerova 2016	Could everyday technology improve access to assessments? A pilot study on the feasibility of screening cognition in people with Parkinson's disease using the Montreal Cognitive Assessment via Internet videoconferencing	MoCA	Comparison against in-person exam	Equivalent
Neurologic	Tarolli 2018	Virtual research visits in individuals with Parkinson disease enrolled in a clinical trial: React-PD study	Parkinson's disease motor and symptom assessments	Comparison against in-person exam	NS
Neurologic	Timpano 2013	Videoconference-based mini mental state examination: a validation study	Italian videoconference-based version of the Mini-Mental State Examination (VMMSE)	Comparison against in-person exam	Equivalent
Neurologic	Vahia 2015	Telepsychiatry for neurocognitive testing in older rural latino adults	Mini-Mental State Examination (MMSE), Hopkins Verbal Learning Test (HVLT)-Revised, Digit Span subtest from the Escala de Inteligencia de Wechsler para Adultos-Tercera Edicion, Letter and Category Fluency, Clock Drawing, Brief Visuospatial Memory Test (revised) (BVMT-R), Ponton-Satz Spanish Naming Test	Comparison against in-person exam	Equivalent
Neurologic	VanHooff 2013	Prehospital unassisted assessment of stroke severity using telemedicine: A feasibility study	Unassisted TeleStroke Scale (UTSS)	Comparison against in-person exam	Equivalent
Neurologic	VanHooff 2013	Unassisted assessment of stroke severity using telemedicine	Unassisted TeleStroke Scale (UTSS)	Comparison against in-person exam	Equivalent
Neurologic	Venkataraman 2014	Virtual visits for Parkinson disease: A case series	components of the Unified Parkinson’s Disease Rating Scale, such as remote analysis of rest tremor, action tremor, finger taps, hand movements, arising from chair, and gait	Feasibility, patient satisfaction	NS
Neurologic	Wadsworth 2016	Remote Neuropsychological Assessment in Rural American Indians with and without Cognitive Impairment	MMSE, Clock Drawing, Digit Span Forward and Backward, Oral Trails, Hopkins Verbal Learning Test-Revised, Letter and Category Fluency, a short form Boston Naming Test	Comparison against in-person exam	Equivalent
Neurologic	Weiner 2011	Videoconference diagnosis and management of Choctaw Indian dementia patients	eyesight, hearing, facial expression, gait and station, coordination, tremor, rapid alternating movements, psychomotor activity, motor tests of executive function	Feasibility	NS
Neurologic	Wong 2012	The Rowland universal dementia assessment scale (RUDAS) as a reliable screening tool for dementia when administered via videoconferencing in elderly post-acute hospital patients	Rowland Universal Dementia Assessment Scale (RUDAS)	Comparison against in-person exam	Equivalent
Neurologic	Wood 2013	Can a low-cost webcam be used for a remote neurological exam?	Kurtzke Expanded Disability Status Scale (EDSS)	Comparison against in-person exam	Equivalent
Neuropsychologic	Appleby 2019	Feasibility of remote assessment of human prion diseases for research and surveillance	Telephone Interview for Cognitive Status (TICS), Medical Research Council (MRC) Prion Disease Rating Scale, Neuropsychiatric Inventory Questionnaire (NPI-Q), neurological exam	Acceptance, ease of use, and satisfaction of teleneurology among subjects and their caregivers	NS
Neuropsychologic	Galusha-Glasscock 2016	Video Teleconference Administration of the Repeatable Battery for the Assessment of Neuropsychological Status	Repeatable Battery for the Assessment of Neuropsychological Status (RBANS)	Comparison against in-person exam	Equivalent
Neuropsychologic	Hildebrand 2004	Feasibility of neuropsychological testing of older adults via videoconference: Implications for assessing the capacity for independent living	Rey Auditory Verbal Learning Test (RAVLT)-T1, RAVLT- T6, RAVLT-LOT, CD, MR, BTA, CWAT, RAVLT-D, VOC	Comparison against in-person exam	Equivalent
Neuropsychologic	Kirkwood 2000	The consistency of neuropsychological assessments performed via telecommunication and face to face	National Adult Reading Test (NART) 11, the Quick Test, Forms 1 and 3, and sections of the Adult Memory and Information Processing Battery (AMIPB) 13, comprising Forms 1 and 2 of the following subtests: Story Recall, List Learning, Figure Recall, Information Processing	Comparison against in-person exam	Equivalent
Neuropsychologic	Vestal 2006	Efficacy of language assessment in Alzheimer's disease: comparing in-person examination and telemedicine.	Picture Description (auditory response version) (Boston Diagnostic Aphasia Examination [BDAE]) (Goodglass et al 2001), Boston Naming Test (BNT) (Goodglass et al 2001), Token Test (Multilingual Aphasia Examination) (Benton et al 2000), Aural Comprehension of Words and Phrases (Benton et al 2000), Controlled Oral Word Association Test (Benton et al 2000)	Comparison against in-person exam	Equivalent
Not specified	Dixon 2008	Virtual visits in a general medicine practice: A pilot study	not specified	Comparison against in-person exam	Inferior
Not specified	Ohta 2017	How Accurate Are First Visit Diagnoses Using Synchronous Video Visits with Physicians?	not specified	Agreement between in-person and telemedicine diagnoses	Equivalent
Psychiatric	Grosch 2015	Video teleconference-based neurocognitive screening in geropsychiatry	Mini-Mental State Examination (MMSE), Clock Drawing Test, Digit Span	Comparison against in-person exam	Equivalent
Psychiatric	Loh 2004	Can patients with dementia be assessed at a distance? The use of Telehealth and standardised assessments	Standardized Mini-Mental State Exam (SMMSE), the Geriatric Depression Scale (GDS)	Comparison against in-person exam	Inferior
Psychiatric	Menon 2001	Evaluation of a portable low cost videophone system in the assessment of depressive symptoms and cognitive function in elderly medically III veterans	Geriatric Depression Scale short version (GDS), Hamilton Depression Scale (HAM-D), Short Portable Mental Status Exam (SPMSE)	Comparison against in-person exam	Equivalent
Psychiatric	Singh 2007	Accuracy of telepsychiatric assessment of new routine outpatient referrals	Not specified	Agreement of DSM-IV diagnoses, risk assessment, non-drug and drug interventions between telemedicine and in-person exams	Equivalent
Skin	Edison 2008	Diagnosis, diagnostic confidence, and management concordance in live-interactive and store-and-forward teledermatology compared to in-person examination	Not specified	Diagnostic and management agreement between in-person and telemedicine exams	Equivalent
Skin	Lesher 1998	Telemedicine evaluation of cutaneous diseases: a blinded comparative study.	Not specified	Agreement in diagnoses between the telemedicine and in-person groups.	Equivalent
Skin	Marchell 2017	Comparing High Definition Live Interactive and Store-and-Forward Consultations to In-Person Examinations	Not specified	Diagnoses, decisions to biopsy, and diagnostic confidence for teledermatology consultations vs in-person	Inferior
Skin	Seghers 2014	A prospective study on the use of teledermatology in psychiatric patients with chronic skin diseases	Not described, but likely only visual inspection.	Comparison against in-person exam	Equivalent
Skin	Smith 2004	Diagnostic accuracy of and patient satisfaction with telemedicine for the follow-up of paediatric burns patients	Scar color, scar thickening, contractures, range of motion, breakdown of the graft site, activity level	Comparison against in-person exam	Equivalent

### Study descriptors

Publication dates of included studies spanned 1993 to 2020 with 2 citations from 1991 to 1995, 4 citations from 1996 to 2000, 6 citations from 2001 to 2005, 10 citations from 2006 to 2010, 23 citations from 2011 to 2015, and 29 citations from 2016 to 2020.

By country, the studies were most frequently conducted in the USA (44/74, 59%), Australia (10/74, 14%), the UK (4/74, 5%), Canada (3/74, 4%), Belgium (2/74, 3%), and Italy (2/74, 3%). By international region, the studies were most frequently conducted in the Americas (47/74, 64%), Europe (12/74, 16%), Oceania (11/74, 15%), and Asia (4/74, 5%).

**Fig. 2:**
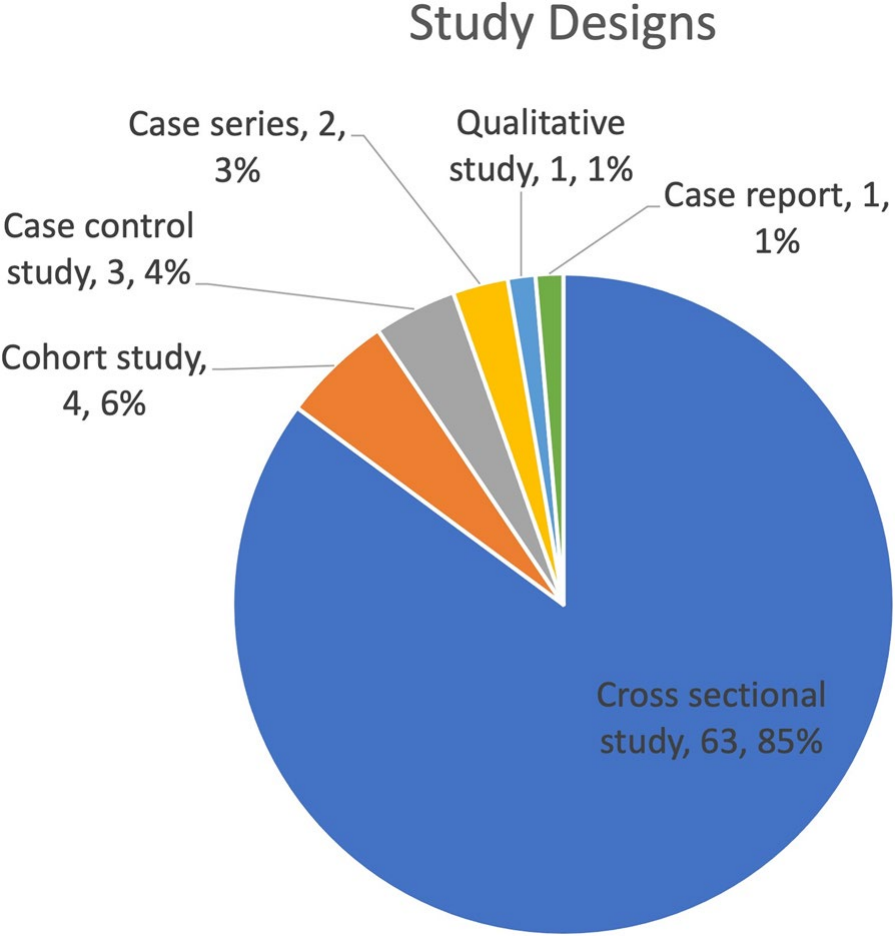
Pie chart showing the frequency of study designs

Eighty-five percent of included studies were cross-sectional studies with the other 15% representing cohort studies, case-control studies, case series, qualitative studies, and case reports (Fig. 2).

Sixty-seven (91%) of the studies were peer-reviewed articles and 7 (9%) were abstracts, presentations, or posters.

### Study population descriptors

Reporting of demographic information, including age, sex, gender, race, ethnicity, and smoking status, was assessed. Fifty-seven (77%) studies reported at least one piece of demographic information as defined here, while 17 (23%) studies did not report any demographic information about the participants.

Fifty-four (73%) studies reported the mean or median age of the study population while 20 (27%) studies did not. Among the studies that reported mean or median age, the average mean or median age of the study population was 57.7 years (SD: 20.4 years). Four (5%) of studies were conducted in pediatric populations only. The median number of participants per study was 27 (IQR: 13-51).

### Health conditions

The most studied medical conditions were Parkinson’s disease (14/74, 19%), Alzheimer’s disease (12/74, 16%), dementia (8/74, 11%), mild cognitive impairment (7/74, 9%), stroke (7/74, 9%), multiple sclerosis (4/74, 5%), and depression (3/74, 4%). Grouping the studied medical conditions more broadly, the most studied disorder classes were neurologic disorders (40/74, 54%), musculoskeletal disorders (8/74, 11%), psychiatric disorders (5/74, 7%), dermatologic disorders (4/74, 5%), urinary disorders (2/74, 3%), pulmonary disorders (2/74, 3%), cardiovascular disorders (2/74, 3%), otolaryngologic disorders (2/74, 3%), hematologic disorders (1/74, 1%), and digestive disorders (1/74, 1%).

### Telemedicine exam

**Fig. 3:**
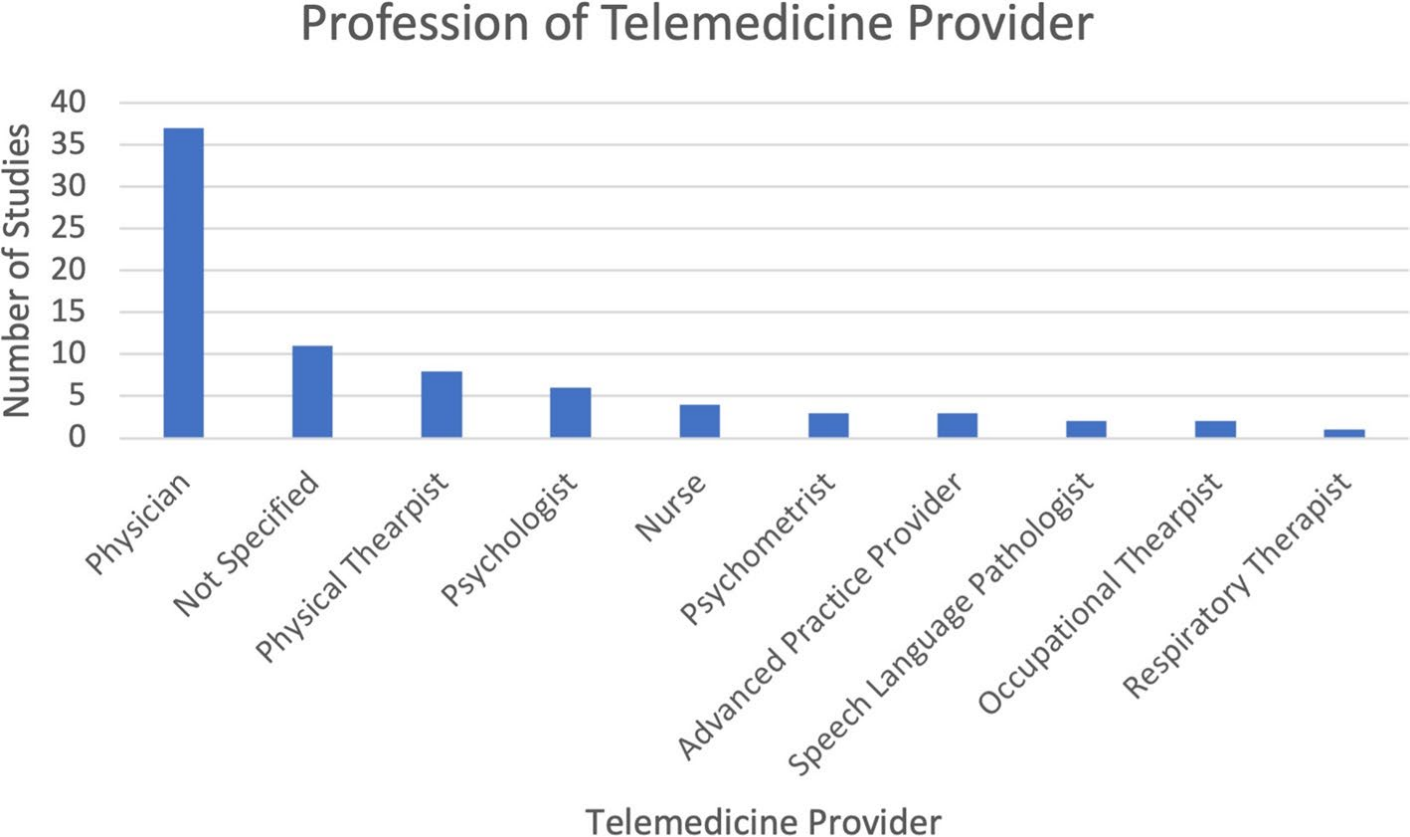
Bar graph showing the professions of the telemedicine providers

The telemedicine physical exam was performed, in descending order of frequency, by physicians (37/74, 50%), physical therapists (8/74, 11%), psychologists (6/74, 8%), nurses (4/74, 5%), psychometrists (3/74, 4%), advanced practice providers (3/74, 4%), speech and language pathologists (2/74, 3%), occupational therapists (2/74, 3%), and respiratory therapists (1/74, 1%) (Fig. 3). In 11 (15%) studies, the profession of the examiner was not specified.

**Fig. 4:**
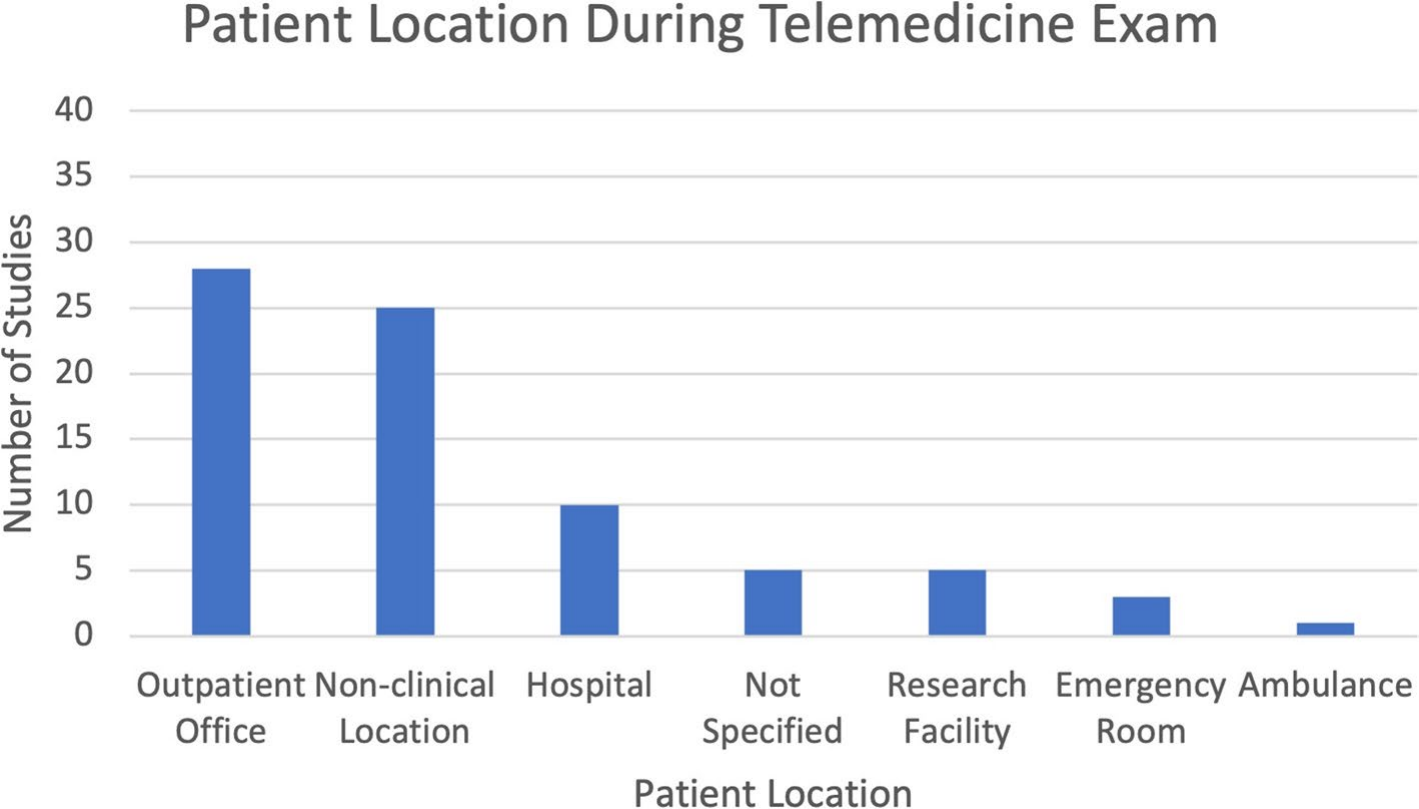
Bar graph showing the patients’ locations during the telemedicine exam

During the telemedicine exam, the patients were most commonly located in outpatient offices (28/74, 38%), homes and other non-clinical settings (25/74, 34%), hospitals (10/74, 14%), research facilities (5/74, 7%), emergency rooms (3/74, 4%), and ambulances (1/74, 1%) (Fig. 4). In 5 (9%) studies, the patient’s location was not specified.

**Fig. 5:**
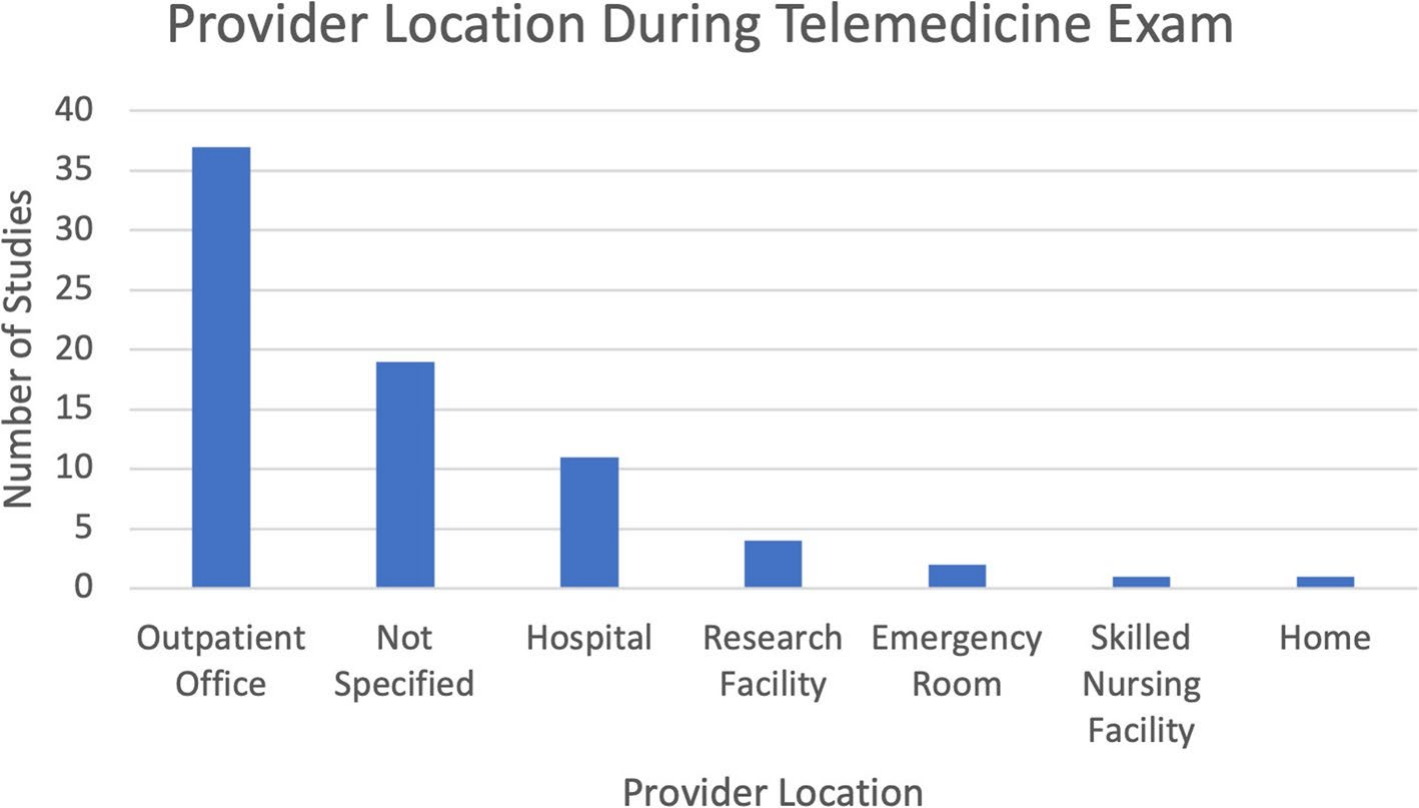
Bar graph showing the providers’ locations during the telemedicine exam

During the telemedicine exam, the providers were most commonly located in outpatient offices (37/74, 50%), hospitals (11/74, 15%), research facilities (4/74, 5%), emergency rooms (2/74, 3%), skilled nursing facilities (1/74, 1%), and at home (1/74, 1%) (Fig. 5). In 19 (26%) studies, the provider’s location was not specified.

On patient side, the telemedicine encounter was most frequently conducted using only computers (49/74, 66%), any devices including computers, tablets, and smartphones (10/74, 14%), only tablets (5/74, 7%), and only smartphones (4/74, 5%). In 7 (9%) studies, the device used by the patient for telecommunications was not specified.

On provider side, the telemedicine encounter was most frequently conducted using only computers (52/74, 70%), only tablets (3/74, 4%), any devices including computers, tablets, and smartphones (2/74, 3%), and only smartphones (1/74, 1%). In 16 (22%) studies, the device used by the provider for telecommunications was not specified.

Although our methodology excluded studies where the exam was facilitated by a physically present health care provider, 14% of studies reported that the telemedicine exam was assisted by an assistant with no medical training and 86% of studies reported that the telemedicine exam was not assisted.

**Fig. 6:**
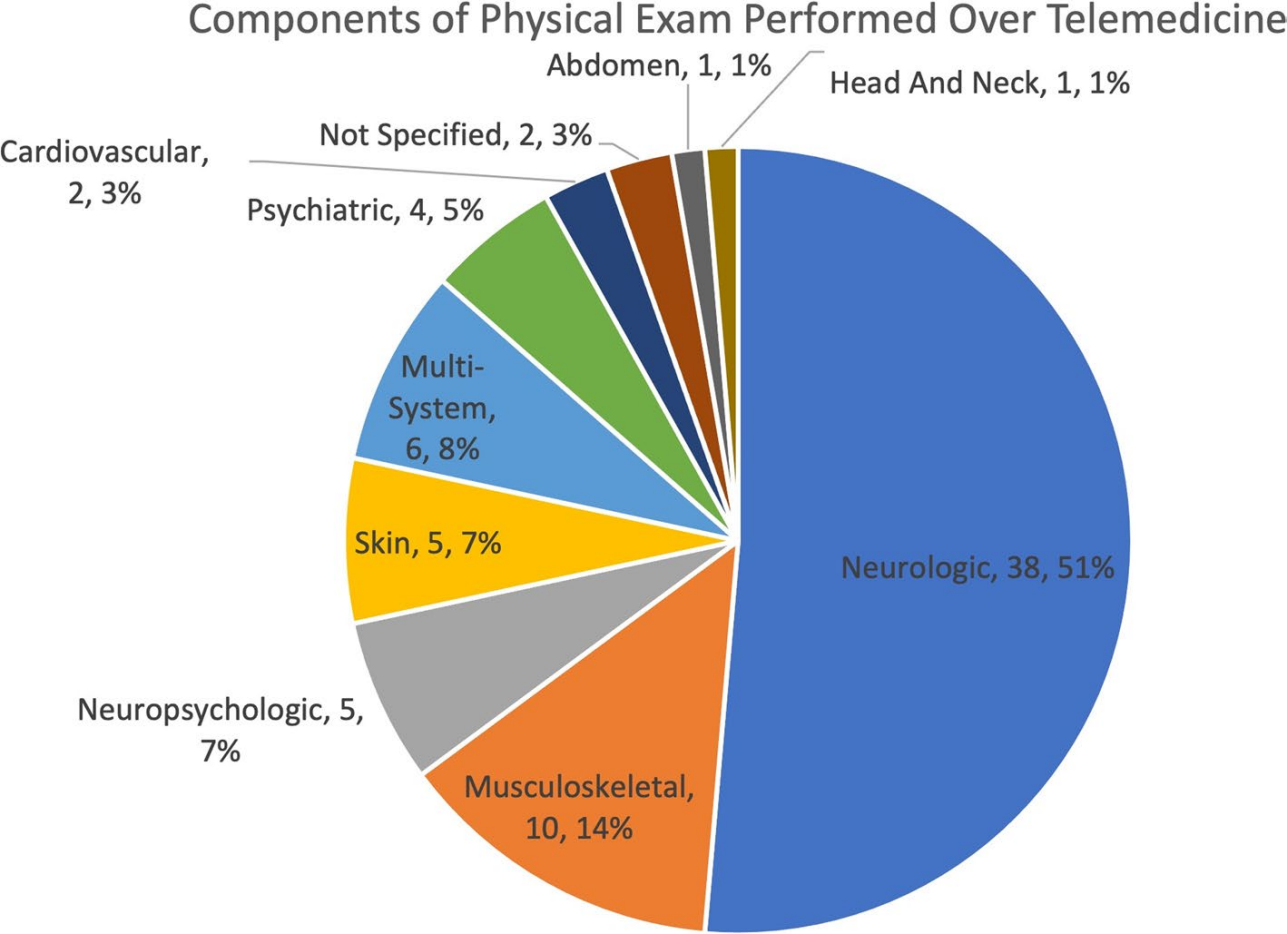
Pie chart showing the components of the telemedicine exam performed in all studies

The most common components of the physical exam performed over telemedicine were neurologic (38/74, 51%), musculoskeletal (10/74, 14%), multi-system (6/74, 8%), neuropsychologic (5/74, 7%), skin (5/74, 7%), psychiatric (4/74, 5%), cardiovascular (2/74, 3%), abdominal (1/74, 1%), and head and neck (1/74, 1%) (Fig. 6). In 2 (3%) studies, the physical exam component was not specified.

In 61 (82%) studies, the outcome of the study was a comparison of the telemedicine exam and the in-person exam. Of these studies, 54 (89%) concluded that the telemedicine exam performed was equivalent to the in-person physical exam whereas 7 (11%) studies [11–17] concluded that the telemedicine exam was inferior to the in-person exam.

## Discussion

This scoping review aimed to synthesize prior research on the unassisted physical exam conducted via real-time audio-video telemedicine.

### Study descriptors

Analysis of the publication dates of included articles demonstrates a steady, shallow increase in publications from 1991 to 2010 followed by a sharp sloping rise in publications from 2011 to 2020. We hypothesize that the change in slope is because the decade from 2010 to 2020 saw telemedicine cross the gap from use by a few visionaries to acceptance by an early majority of pragmatists [18]. Though our study does not capture the literature from the COVID era, we expect the rise of publications on the telemedicine physical exam to accelerate even more rapidly as COVID catapulted telemedicine to the forefront of care delivery.

The English literature demonstrated a skew toward the studies conducted in the USA, which accounted for 59% of publications. Notably, none of the included studies were conducted in Africa, though all the other major international regions—the Americans, Europe, Oceania, and Asia—were represented. Telemedicine is an important component in addressing healthcare needs and access in Africa [8, 19], and increased attention to the telemedicine physical exam in this region is needed to address this gap in the literature.

Evaluated by study design, the body of literature is skewed toward a low level of evidence, with cross-sectional studies representing 86% of included studies. Notably, there were no randomized controlled trials that compared the telemedicine exam and in-person physical exam.

### Study population descriptors

Almost a quarter (23%) of studies did not report demographic information about the study participants. Improvements in demographic reporting in this field would contribute toward a collective responsibility to seek clarity and transparency in the representativeness and diversity of study participants [20] and is especially important in the context of recent research showing disparities in telehealth utilization [21].

The sample sizes of all included studies were relatively small with a median size of 27 participants among included studies and future studies may benefit from larger sample sizes. The bulk of the literature focuses on the telemedicine physical exam in the adult population, with only 5% of studies conducted specifically in the pediatric population. Given the increased adoption of telemedicine by pediatricians and pediatric medical and surgical specialists [22], evaluations focused on the telemedicine exam in children are an important direction for future research.

### Telemedicine exam

Though most telemedicine exams in included studies were conducted by physicians (50%), our research demonstrates a wide variety of other healthcare providers including nurses, physical therapists, and speech language pathologists, which fits well with our perception that telemedicine will be a way of providing evaluations and delivering health care across the medical spectrum.

In the included studies, the two most common patient locations were outpatient offices (38%) and homes and other non-clinical settings (34%). In many studies, patients were in the outpatient offices for research convenience or access to the non-portable telecommunications equipment supplied by researchers. Also, the most studied device used for telemedicine encounters by both providers and patients was the computer. Portable devices such as tablets and smartphones were only used by patients in 12% of studies and by providers in 5% of studies. As modern-day telemedical examination increasingly moves to the home and to mobile devices, it will be crucial for future studies to further examine the efficacy of the physical exam in a home setting on mobile devices [18].

The studies of the virtual neurological exam, in conjunction with the musculoskeletal, neuropsychologic, and skin exams, comprise 79% of the literature and are highly overrepresented compared to other exam areas. The overrepresentation of these physical exam components may reflect the early adoption of telemedicine in the corresponding medical fields. For example, telestroke services, first described in 1999 [23], have been integrated in stroke systems of care for more than 10 years [24]. The success of the neurologic exam in telestroke may have subsequently spurred the application of telemedicine for a range of other neurologic conditions such as dementia, epilepsy, movement disorders, and multiple sclerosis [25]. In addition, the prevalence of studies on the neuropsychologic exam may reflect the ease of transitioning a verbal exam to the telemedicine setting. Nonetheless, common exam maneuvers performed over telemedicine are not limited to these areas. An analysis of the American Medical Association’s 2016 Physician Practice Benchmark Survey shows that the specialists using telemedicine the most to interact with patients are radiologists, psychiatrists, and cardiologists. The subspecialties most often using video-conferencing-based telemedicine to interact with patients or other health care professionals are emergency medicine, psychiatry, and pathology [26]. Given the range of medical specialists practicing telemedicine, future research should aim to expand the range of exam components studied with particular attention to less researched exam areas.

### Limitations

There are several limitations to this scoping review. First, the literature search was conducted on March 17, 2020. As a result, our review does not include articles on the telemedicine exam from the COVID era. Given the meteoritic rise in telemedicine use spurred by region-wide lockdowns and concerns over COVID transmission, a follow-up review including articles on the telemedicine exam in the COVID era would be an important future research direction. Second, our review focuses on the most basic form of a telemedicine physical exam—one without the assistance of technology and trained medical personnel on the patient side. We chose to focus on an unassisted exam to investigate the exam components that could be performed under the widest variety of clinical practice settings, but we acknowledge that assistive technology will be important in facilitating an accurate and effective physical exam over telemedicine in the future. Third, though many publications in our review use comparison between the telemedicine physical exam and the in-person exam as a primary outcome, in practice, outcomes with direct impact on patient care may be more meaningful. After all, the goal of the telemedicine examination is not to directly translate the in-person exam, but to create a set of virtual exam maneuvers that provide the highest degree of actionable information to the clinician. Lastly, our restriction on English language articles may have excluded relevant studies in other languages, as telemedicine is a global phenomenon.

## Conclusions

In conclusion, the current research on an unassisted physical exam performed over synchronous audiovisual telemedicine is focused on the neurologic, musculoskeletal, neuropsychologic, and skin exam components. The majority (89%) of studies that directly compared the virtual exam to the in-person exam concluded that the telemedicine exam was equivalent to the in-person exam. Research evaluating the virtual physical exam is at an early stage of maturity with current limitations including body of literature mostly comprised of cross-sectional studies representing a low level of evidence, incomplete demographic reporting, paucity of studies focusing on the pediatric exam, and a minority of studies looking at exams performed on mobile devices and in home settings. Future research in this area should focus on expanding the range of telemedicine exam maneuvers studied and evaluating the exam in the most relevant settings, which for telemedicine is trending toward exams through mobile devices and in patients’ homes. In the long term, this review aims to guide the development of a virtual physical exam that provides the highest degree of clinically relevant information to the clinician.

## Supplementary Information

**Additional file 1.** PRISMA-ScR Checklist

**Additional file 2: Supplementary Appendix 1.** Ovid MEDLINE search strategy. **Supplementary Appendix 2.** Data Extraction Definitions. **Supplementary Appendix 3.** Citations for all included articles.

## Data Availability

The datasets used and/or analyzed during the current study are available from the corresponding author on reasonable request.

## References

[1] Portnoy J, Waller M, Elliott T (2020). Telemedicine in the era of COVID-19. J Allergy Clin Immunol Pract.

[2] Telemedicine: what should the post-pandemic regulatory and payment landscape look like? | Commonwealth Fund. https://www.commonwealthfund.org/publications/issue-briefs/2020/aug/telemedicine-post-pandemic-regulation. Accessed 18 Nov 2021.

[3] Telehealth and patient safety during the COVID-19 response | PSNet. https://psnet.ahrq.gov/perspective/telehealth-and-patient-safety-during-covid-19-response. Accessed 18 Nov 2021.

[4] NQF: creating a framework to support measure development for telehealth. https://www.qualityforum.org/Publications/2017/08/Creating_a_Framework_to_Support_Measure_Development_for_Telehealth.aspx. Accessed 18 Nov 2021.

[5] The evidence base for telehealth: reassurance in the face of rapid expansion during the COVID-19 pandemic | Effective Health Care (EHC) Program. https://effectivehealthcare.ahrq.gov/products/telehealth-expansion/white-paper. Accessed 18 Nov 2021. 32479040

[6] Hayden EM, Davis C, Clark S, et al. Telehealth in emergency medicine: a consensus conference to map the intersection of telehealth and emergency medicine. Acad Emerg Med. 2021. 10.1111/ACEM.14330. 10.1111/acem.14330 PMC11150898 34245649

[7] Verghese A, Horwitz RI (2009). In praise of the physical examination. BMJ..

[8] Adepoju P (2020). Africa turns to telemedicine to close mental health gap. Lancet Digit Heal.

[9] Ansary AM, Martinez JN, Scott JD (2021). The virtual physical exam in the 21st century. J Telemed Telecare.

[10] Tricco AC, Lillie E, Zarin W (2018). PRISMA extension for scoping reviews (PRISMA-ScR): Checklist and explanation. Ann Intern Med.

[11] Stillerova T, Liddle J, Gustafsson L, Lamont R, Silburn P. Remotely assessing symptoms of Parkinson’s disease using videoconferencing: a feasibility study. Neurol Res Int. 2016;2016. 10.1155/2016/4802570. 10.1155/2016/4802570 PMC5220499 28116158

[12] Schoenfeld AJ, Davies JM, Marafino BJ (2016). Variation in quality of urgent health care provided during commercial virtual visits. JAMA Intern Med.

[13] Marchell R, Locatis C, Burges G, Maisiak R, Liu WL, Ackerman M (2017). Comparing high definition live interactive and store-and-forward consultations to in-person examinations. Telemed J E Health.

[14] Dixon RF, Stahl JE (2008). Virtual visits in a general medicine practice: a pilot study. Telemed J E Health.

[15] Montani C, Billaud N, Tyrrell J (1997). Psychological impact of a remote psychometric consultation with hospitalized elderly people. J Telemed Telecare.

[16] Loh PK, Ramesh P, Maher S, Saligari J, Flicker L, Goldswain P (2004). Can patients with dementia be assessed at a distance? The use of Telehealth and standardised assessments. Intern Med J.

[17] Akhtar M, Van Heukelom PG, Ahmed A (2018). Telemedicine physical examination utilizing a consumer device demonstrates poor concordance with in-person physical examination in emergency department patients with sore throat: a prospective blinded study. Telemed J E Health.

[18] Dorsey ER, Topol EJ (2020). Telemedicine 2020 and the next decade. Lancet..

[19] Wamala DS, Augustine K (2013). A meta-analysis of telemedicine success in Africa. J Pathol Inform.

[20] Rubin E (2021). Striving for Diversity in Research Studies. N Engl J Med.

[21] Lame M, Leyden D, Platt SL (2021). Geocode maps spotlight disparities in telehealth utilization during the COVID-19 pandemic in New York City. Telemed J E Health.

[22] Hall RW, Dehnel PJ, Alexander JJ (2015). Telemedicine: pediatric applications. Pediatrics..

[23] Levine SR, Gorman M (1999). “Telestroke”: the application of telemedicine for stroke. Stroke..

[24] Wechsler LR, Demaerschalk BM, Schwamm LH (2017). Telemedicine quality and outcomes in stroke: a scientific statement for healthcare professionals from the American Heart Association/American Stroke Association. Stroke..

[25] Hatcher-Martin JM, Adams JL, Anderson ER (2020). Telemedicine in neurology: Telemedicine Work Group of the American Academy of Neurology update. Neurology..

[26] Kane CK, Gillis K (2018). The use of telemedicine by physicians: still the exception rather than the rule. Health Aff (Millwood).

