# Supplementary material for: A scoping review of the unassisted physical exam conducted over synchronous audio-video telemedicine
Source: Syst Rev. 2022 Oct 13;11:219. doi: 10.1186/s13643-022-02085-1 (PMC9559902; doi:10.1186/s13643-022-02085-1)
Supplement: Supplementary file 2 — Additional file 2: Supplementary Appendix 1. Ovid MEDLINE search strategy. Supplementary Appendix 2. Data Extraction Definitions. Supplementary Appendix 3. Citations for all included articles. [file 13643_2022_2085_MOESM2_ESM.docx]

**Supplemental Appendix 1**

OVID MEDLINE® ALL and Cochrane Library Search (1990 – March 17, 2020)

Run on 3/17/20

1. Telemedicine/ or Remote Consultation/ or Videoconferencing/

2. Physical Examination/ or Self-Examination/ or Diagnostic Techniques, Otological/ or Hearing Tests/ or Breast Self-Examination/ or Diagnostic Techniques, Cardiovascular/ or Diagnostic Techniques, Digestive System/ or Digital Rectal Examination/ or Diagnostic Techniques, Endocrine/ or Gynecological Examination/ or "Mental Status and Dementia Tests"/ or Neurologic Examination/ or Diagnostic Techniques, Respiratory System/ or Diagnostic Techniques, Urologic/ or Diagnostic Techniques, Ophthalmological/ or Vital Signs/ or Blood Pressure/ or Body Temperature/ or Heart Rate/ or Respiratory Rate/ or Heart Rate, Fetal/ or Body Height/ or Body Weight/ or Body Mass Index/ or Apgar Score/ or Auscultation/ or Heart Auscultation/ or Blood Pressure Determination/ or Somatotypes/ or Physical Appearance, Body/ or Facies/ or Gait/ or Walking Speed/ or Heart Rate Determination/ or Muscle Strength/ or Palpation/ or Percussion/ or Reflex/ or Skin Pigmentation/ or Range of Motion, Articular/ or Visual Acuity/ or Visual Field Test/ or Central Venous Pressure/ or Edema/

3. (telemedicine or tele medicine or tele-medicine or mobile health or mHealth or m-health or telehealth or tele health or tele-health or eHealth or e-health or remote consultation* or teleconsultation* or tele-consultation* or tele consultation* or video consultation* or virtual consultation* or televisit* or tele-visit* or tele visit* or eVisit* or e-visit* or video visit* or remote visit* or virtual visit* or video encounter* or remote encounter* or virtual encounter* or telediagnos* or tele-diagnos* or tele diagnos* or remote diagnos* or virtual diagnos* or video diagnos* or videoconferenc* or video conferenc* or real-time video or synchronous video or telecardiolog* or tele-cardiolog* or tele cardiolog* or teledermatolog* or tele-dermatolog* or tele dermatolog*).mp. [mp=title, abstract, original title, name of substance word, subject heading word, floating sub-heading word, keyword heading word, organism supplementary concept word, protocol supplementary concept word, rare disease supplementary concept word, unique identifier, synonyms]

4. (physical exam* or "physical exam* and diagnos*" or virtual exam* or remote exam* or video exam* or medical exam* or health exam* or routine exam* or clinical exam* or self exam* or self-exam* or auditory system exam* or otologic exam* or ear exam* or otological exam* or hearing test* or acoustic test* or auditory test* or biliary tract exam* or bladder exam* or bone exam* or breast exam* or breast self-exam* or breast self exam* or cardiovascular system exam* or cardiovascular diagnostic technique* or cardiovascular diagnostic technic* or digestive system exam* or digestive system diagnostic technique* or digestive system diagnostic technic* or digital rectal exam* or digital rectal palpation* or rectal digital exam* or rectal touch* or endocrine system exam* or endocrine diagnostic technique* or endocrine diagnostic technic* or eye exam* or ocular exam* or ENT exam* or ORL exam* or otorhinolaryngologic exam* or HEENT exam* or "ear, nose, and throat exam*" or nose exam* or nasal exam* or esophagus exam* or gastrointestinal tract exam* or GI tract exam* or genital system exam* or gynecological exam* or gynaecological exam* or gynaecologic exam* or gynecologic exam* or gynecologic investigation* or hepatobiliary system exam* or intestine exam* or joint exam* or kidney exam* or liver exam* or lung exam* or pulmonary exam* or lymphatic system exam* or male genital system exam* or General Practitioner Assessment of Cognition or GPCOG or Montreal Cognitive Assessment* or Mental Status Exam* or Mental Status Test* or Neurocognitive Test* or Neurobehavioral Cognitive Status Exam* or COGNISTAT or Unified Parkinson's Disease Rating Scale or UPDRS Panel or Mini Mental State Exam* or Folstein Mini-Mental State Exam* or MMSE* or Mini Mental State Exam* or Mini Mental Status Exam* or Mini-Cog or Clinical Dementia Rating or Clinical Dementia Rating Scale or Dementia Rating Scale or MicroCog or Cognitive Assessment Screening Instrument or dementia test* or dementia assessment* or mouth exam* or oral exam* or oral cavity exam* or musculoskeletal system exam* or neuro exam* or neurologic exam* or pancreas exam* or parasite exam* or parathyroid gland exam* or parathyroid exam* or vaginal exam* or pelvic exam* or periodic medical exam* or medical surveillance or periodic checkup* or periodic exam* or periodic health exam* or periodontal exam* or pharynx exam* or throat exam* or respiratory tract exam* or respiratory system diagnostic technique* or respiratory system exam* or salivary gland exam* or sensory system exam* or sensory exam* or skin exam* or dermatologic exam* or spleen exam* or stomach exam* or testicular self exam* or thorax exam* or thyroid gland exam* or thyroid exam* or urogenital system exam* or urologic exam* or urologic diagnostic technique* or visual system exam* or ophthalmological diagnostic technique* or ophthalmological diagnostic technics* or wound assessment* or wound classification* or wound measurement* or wound exam* or vital sign* or blood pressure* or blood tension* or intravascular pressure* or normotension or vascular pressure* or diastolic pressure* or systolic pressure* or pulse pressure* or body temperature* or body heat* or normothermia or organ temperature* or heart rate* or pulse rate* or cardiac chronotrop* or heart rate control or cardiac frequency or cardiac rate* or heart frequency or ventricle rate* or pulse or respirat* rate* or breathing rate* or breathing frequency or respirat* frequency or fetal heart rate* or fetus heart rate* or foetus heart rate* or foetal heart rate* or body height* or body length* or stature or body weight* or total body weight* or body mass index or BMI or body ban mass or Quetelet* index or head circumference* or cranial circumference* or apgar score* or apgar classification* or apgar coefficient* or apgar index or apgar rating* or apgar scale* or apgar test* or neonatal apgar or auscultation* or heart auscultation or cardiac auscultation or lung auscultation or pulmonary auscultation or blood pressure measurement* or blood pressure determination* or blood pressure recording* or direct blood pressure measurement* or BP measurement* or somatyp* or body build* or body typ* or endomorph* or mesomorph* or ectomorph* or physical appearance* or outward appearance* or body physical appearance* or facial appearance* or facies* or facial feature* or biped gait* or gait analys* or gait training* or walking pattern* or gait* or walking speed* or gait speed* or gait velocit* or stride speed* or stride velocit* or walk speed* or walk velocit* or walking rate* or walking velocit* or walking pace* or heart rate determination* or heart rate measurement* or pulse rate determination* or pulse rate measurement* or muscle strength* or muscular strength* or muscle power or muscular power or muscular force or muscle force or palpation* or percussion* or reflex* or reflex movement* or reflex activit* or reflex response* or skin pigmentation* or cutaneous pigmentation* or skin pigment* or skin tone* or skin color* or articular range of motion or joint range of motion or range of motion or ROM or visual acuity or visual resolution or visual sharpness or visual field test* or visual field exam* or perimetry or central venous pressure* or CVP* or jugular vein pressure* or JVP* or jugular venous pressure* or tissue swelling or venous edema or venous oedema or oedema or hydrops or anasarca).mp. [mp=title, abstract, original title, name of substance word, subject heading word, floating sub-heading word, keyword heading word, organism supplementary concept word, protocol supplementary concept word, rare disease supplementary concept word, unique identifier, synonyms]

5. 2 or 4

6. 1 or 3

7. 5 and 6

8. limit 7 to yr="1990 -Current"

OVID EMBASE (1990 – March 17, 2020) Search

Run on 3/17/20

1. telemedicine/ or telehealth/ or teleconsultation/ or telediagnosis/ or videoconferencing/ or telecardiology/ or teledermatology/

2. physical examination/ or medical examination/ or clinical examination/ or self examination/ or auditory system examination/ or hearing test/ or biliary tract examination/ or bladder examination/ or bone examination/ or breast examination/ or breast self examination/ or cardiovascular system examination/ or digestive system examination/ or digital rectal examination/ or endocrine system examination/ or eye examination/ or ENT examination/ or esophagus examination/ or gastrointestinal tract examination/ or genital system examination/ or gynecological examination/ or hepatobiliary system examination/ or intestine examination/ or joint examination/ or kidney examination/ or liver examination/ or lung exam/ or lymphatic system examination/ or male genital system examination/ or dementia assessment/ or mouth examination/ or musculoskeletal system examination/ or neurologic examination/ or pancreas examination/ or parasite examination/ or parathyroid gland examination/ or pelvic examination/ or periodic medical examination/ or periodontal examination/ or pharynx examination/ or respiratory tract examination/ or salivary gland examination/ or sensory system examination/ or skin examination/ or spleen examination/ or stomach examination/ or testicular self examination/ or thorax examination/ or thyroid gland examination/ or urogenital system examination/ or urologic examination/ or visual system examination/ or wound assessment/ or vital sign/ or blood pressure/ or body temperature/ or heart rate/ or breathing rate/ or fetus heart rate/ or body height/ or body weight/ or body mass/ or head circumference/ or Apgar score/ or auscultation/ or heart auscultation/ or lung auscultation/ or blood pressure measurement/ or somatotype/ or physical appearance/ or facies/ or gait/ or walking speed/ or heart rate measurement/ or muscle strength/ or palpation/ or percussion/ or reflex/ or skin pigmentation/ or "joint characteristics and functions"/ or visual acuity/ or perimetry/ or central venous pressure/ or edema/

3. (telemedicine or tele medicine or tele-medicine or mobile health or mHealth or m-health or telehealth or tele health or tele-health or eHealth or e-health or remote consultation* or teleconsultation* or tele-consultation* or tele consultation* or video consultation* or virtual consultation* or televisit* or tele-visit* or tele visit* or eVisit* or e-visit* or video visit* or remote visit* or virtual visit* or video encounter* or remote encounter* or virtual encounter* or telediagnos* or tele-diagnos* or tele diagnos* or remote diagnos* or virtual diagnos* or video diagnos* or videoconferenc* or video conferenc* or real-time video or synchronous video or telecardiolog* or tele-cardiolog* or tele cardiolog* or teledermatolog* or tele-dermatolog* or tele dermatolog*).mp. [mp=title, abstract, heading word, drug trade name, original title, device manufacturer, drug manufacturer, device trade name, keyword, floating subheading word, candidate term word]

4. (physical exam* or "physical exam* and diagnos*" or virtual exam* or remote exam* or video exam* or medical exam* or health exam* or routine exam* or clinical exam* or self exam* or self-exam* or auditory system exam* or otologic exam* or ear exam* or otological exam* or hearing test* or acoustic test* or auditory test* or biliary tract exam* or bladder exam* or bone exam* or breast exam* or breast self-exam* or breast self exam* or cardiovascular system exam* or cardiovascular diagnostic technique* or cardiovascular diagnostic technic* or digestive system exam* or digestive system diagnostic technique* or digestive system diagnostic technic* or digital rectal exam* or digital rectal palpation* or rectal digital exam* or rectal touch* or endocrine system exam* or endocrine diagnostic technique* or endocrine diagnostic technic* or eye exam* or ocular exam* or ENT exam* or ORL exam* or otorhinolaryngologic exam* or HEENT exam* or "ear, nose, and throat exam*" or nose exam* or nasal exam* or esophagus exam* or gastrointestinal tract exam* or GI tract exam* or genital system exam* or gynecological exam* or gynaecological exam* or gynaecologic exam* or gynecologic exam* or gynecologic investigation* or hepatobiliary system exam* or intestine exam* or joint exam* or kidney exam* or liver exam* or lung exam* or pulmonary exam* or lymphatic system exam* or male genital system exam* or General Practitioner Assessment of Cognition or GPCOG or Montreal Cognitive Assessment* or Mental Status Exam* or Mental Status Test* or Neurocognitive Test* or Neurobehavioral Cognitive Status Exam* or COGNISTAT or Unified Parkinson's Disease Rating Scale or UPDRS Panel or Mini Mental State Exam* or Folstein Mini-Mental State Exam* or MMSE* or Mini Mental State Exam* or Mini Mental Status Exam* or Mini-Cog or Clinical Dementia Rating or Clinical Dementia Rating Scale or Dementia Rating Scale or MicroCog or Cognitive Assessment Screening Instrument or dementia test* or dementia assessment* or mouth exam* or oral exam* or oral cavity exam* or musculoskeletal system exam* or neuro exam* or neurologic exam* or pancreas exam* or parasite exam* or parathyroid gland exam* or parathyroid exam* or vaginal exam* or pelvic exam* or periodic medical exam* or medical surveillance or periodic checkup* or periodic exam* or periodic health exam* or periodontal exam* or pharynx exam* or throat exam* or respiratory tract exam* or respiratory system diagnostic technique* or respiratory system exam* or salivary gland exam* or sensory system exam* or sensory exam* or skin exam* or dermatologic exam* or spleen exam* or stomach exam* or testicular self exam* or thorax exam* or thyroid gland exam* or thyroid exam* or urogenital system exam* or urologic exam* or urologic diagnostic technique* or visual system exam* or ophthalmological diagnostic technique* or ophthalmological diagnostic technics* or wound assessment* or wound classification* or wound measurement* or wound exam* or vital sign* or blood pressure* or blood tension* or intravascular pressure* or normotension or vascular pressure* or diastolic pressure* or systolic pressure* or pulse pressure* or body temperature* or body heat* or normothermia or organ temperature* or heart rate* or pulse rate* or cardiac chronotrop* or heart rate control or cardiac frequency or cardiac rate* or heart frequency or ventricle rate* or pulse or respirat* rate* or breathing rate* or breathing frequency or respirat* frequency or fetal heart rate* or fetus heart rate* or foetus heart rate* or foetal heart rate* or body height* or body length* or stature or body weight* or total body weight* or body mass index or BMI or body ban mass or Quetelet* index or head circumference* or cranial circumference* or apgar score* or apgar classification* or apgar coefficient* or apgar index or apgar rating* or apgar scale* or apgar test* or neonatal apgar or auscultation* or heart auscultation or cardiac auscultation or lung auscultation or pulmonary auscultation or blood pressure measurement* or blood pressure determination* or blood pressure recording* or direct blood pressure measurement* or BP measurement* or somatyp* or body build* or body typ* or endomorph* or mesomorph* or ectomorph* or physical appearance* or outward appearance* or body physical appearance* or facial appearance* or facies* or facial feature* or biped gait* or gait analys* or gait training* or walking pattern* or gait* or walking speed* or gait speed* or gait velocit* or stride speed* or stride velocit* or walk speed* or walk velocit* or walking rate* or walking velocit* or walking pace* or heart rate determination* or heart rate measurement* or pulse rate determination* or pulse rate measurement* or muscle strength* or muscular strength* or muscle power or muscular power or muscular force or muscle force or palpation* or percussion* or reflex* or reflex movement* or reflex activit* or reflex response* or skin pigmentation* or cutaneous pigmentation* or skin pigment* or skin tone* or skin color* or articular range of motion or joint range of motion or range of motion or ROM or visual acuity or visual resolution or visual sharpness or visual field test* or visual field exam* or perimetry or central venous pressure* or CVP* or jugular vein pressure* or JVP* or jugular venous pressure* or tissue swelling or venous edema or venous oedema or oedema or hydrops or anasarca).mp. [mp=title, abstract, heading word, drug trade name, original title, device manufacturer, drug manufacturer, device trade name, keyword, floating subheading word, candidate term word]

5. 2 or 4

6. 1 or 3

7. 5 and 6

8. limit 7 to yr="1990 -Current"

**Supplemental Appendix 2**

Data Extraction Definitions.

| **Field** | **Definition** |
| --- | --- |
| **General** |  |
| Title | Title of the study. |
| Author | First and last names of the authors. |
| Journal | Journal in which the study was published. |
| Year of Publication | Year of publication of the study. |
| Country | Country in which study was conducted. |
| Region | Region in which study was conducted categorized by the United Nations geoscheme. One of the following:   1. Africa 2. Americas 3. Asia 4. Europe 5. Oceania 6. Multi-region 7. Not specified 8. Other |
| Study Design | Selected from:   - Randomised controlled trial - Non-randomised experimental study - Cohort study - Cross sectional study - Case control study - Qualitative research - Prevalence study - Case series - Case report - other |
| Publication Format | Selected from:   - Peer-reviewed paper - Abstract (presentation or poster) - other |
| **Participants** |  |
| Age of Study Population Min | Minimum age of study population. |
| Age of Study Population Max | Maximum age of study population. |
| Age of Study Population Average | Average age of study population. |
| Population Description | Description of the population from which data was collected. |
| Number of Participants | The number of participants in the study. |
| Reported population demographics | Whether the study reported the demographics of the sample population (yes/no). Select “‘yes” if age, sex, gender, race, ethnicity, smoking status, etc. |
| Target Health Conditions | The health condition of interest as stated by the study authors or inferred by reviewers. Select “not specified”  if no condition was stated or inferable. |
| **Telemedicine and Physical Exam** |  |
| Telemedicine communications medium for patient | The category of communications device used for the audiovisual synchronous telemedicine link on the patient side. Selected from:   - computer - smartphone - Tablet - Not specified - other |
| Telemedicine communications medium for examiner | The category of communications device used for the audiovisual synchronous telemedicine link on the examiner side. Selected from:   - computer - smartphone - Tablet - Not specified - other |
| Location of Patient | Physical location of the patient during the performance of the virtual exam. Selected from:   - Patient’s private location - Outpatient office - Research facility - Hospital - Emergency department - Not specified - other |
| Location of Examiner | Physical location of the examiner during the performance of the virtual exam.   - Home office - Outpatient office - Research facility - Hospital - Emergency department - Not specified - other |
| Profession of Examiner | Profession of the healthcare provider performing the virtual physical exam. Selected from:   - Physician - Nurse - Advanced practice provider (NP or PA) - Physical therapist - Not specified - other |
| Speciality/Field of Examiner | Medical field or specialty of the healthcare provider performing the virtual physical exam (neurology, dermatology, etc). |
| Category of Exam Performed | Portions of the physical exam were categorized into the following categories. If the exam encompassed multiple system categories, select “other” and list all systems separated by commas. If exam is neuropsychiatric, please select “other” and type “neuropsychiatric”.   - Vitals signs - Skin - Head and neck - Thorax, back, lungs - Breasts - heart/cardiovascular - Abdomen - Upper and lower extremities - Genital and rectal - Neurologic - Psychologic - Not specified - other |
| Specific Exam Maneuvers Performed | List the specific physical exam maneuvers performed separated by commas. |
| Assistance by Nonmedical Personnel | Whether the performance of the physical exam assisted by nonmedical personnel on the patient side (such as family members) (yes/no). |
| Outcome Measures | What was the primary outcome measure of the study relating to the telemedicine physical exam? |
| Positive or Negative Result | If the virtual physical exam is compared to an in-person exam or another reference standard relative to a measurable outcome, select “positive” if the virtual physical exam is as good or better than reference standard. If no obvious comparator was mentioned, select N/A. |
| Result - Free Text | What was the primary finding of the study? |
| **Study Reporting** |  |
| Stated Study Limitations | Whether the article discussed any potential limitations of the research (yes/no). |
| Study limitations - Free Text | Reviewers included article quotations and summaries in this section to capture different reported limitations. Reviewers attempted to only extract free text regarding each specific type of limitation once. |
| Stated Rationale for telemedicine exam | Whether the article stated any reasons for using a telemedicine physical exam instead of an in-person exam (yes/no). |
| Rationale for Telemedicine Exam - Free Text | Reviewers included article quotations and summaries in this section to capture different rationales for using telemedicine. Reviewers attempted to only extract free text regarding each specific type of rationale once. |
| Reviewer Notes | Optional free text notes by reviewer. |

**Supplemental Appendix 3**

Citations for all included articles.

1. A. A, M.T. B, K.C. D, et al. A feasibility study of conducting the Montreal Cognitive Assessment remotely in individuals with movement disorders. *Health Informatics J*. 2016;22(2 PG-304-311):304-311. doi:http://dx.doi.org/10.1177/1460458214556373

2. A. L, A. S, B. L, et al. Dementia Care Comes Home: Patient and Caregiver Assessment via Telemedicine. *Gerontologist*. 2017;57(5 PG-85-93):e85-e93. doi:http://dx.doi.org/10.1093/geront/gnw206

3. A.C. S, K.H. S, M.T.W. C, S.K. N, M.B.Y. T. A prospective study on the use of teledermatology in psychiatric patients with chronic skin diseases. *Ann Acad Med Singapore*. 2014;43(9 SUPPL. 1 PG-S333):S333. http://www.annals.edu.sg/pdf/43VolNo9Sep2014/SHBC2014_Final.pdf NS  -.

4. A.C. S, R. K, J. M, D. B, P. O, R. W. Diagnostic accuracy of and patient satisfaction with telemedicine for the follow-up of paediatric burns patients. *J Telemed Telecare*. 2004;10(4 PG-193-198):193-198. doi:http://dx.doi.org/10.1258/1357633041424449

5. A.J. S, J.M. D, B.J. M, et al. Variation in quality of urgent health care provided during commercial virtual visits. *JAMA Intern Med*. 2016;176(5 PG-635-642):635-642. doi:http://dx.doi.org/10.1001/jamainternmed.2015.8248

6. Abel KC, Baldwin K, Chuo J, et al. Can Telemedicine Be Used for Adolescent Postoperative Knee Arthroscopy Follow-up? *JBJS J Orthop Physician Assist*. October 2017:1. doi:10.2106/jbjs.jopa.17.00014

7. B.R. R, P. T, R. B, T.G. R. Physiotherapy assessment and diagnosis of musculoskeletal disorders of the knee via telerehabilitation. *J Telemed Telecare*. 2017;23(1 PG-88-95):88-95. doi:http://dx.doi.org/10.1177/1357633X15627237

8. B.S. A, K. G, D.D. R, A. B, M.L. C, S. M. Feasibility of remote assessment of human prion diseases for research and surveillance. *Dement Geriatr Cogn Disord*. 2019;47(1-2 PG-79-90):79-90. doi:http://dx.doi.org/10.1159/000497055

9. Bove R, Bevan C, Crabtree E, et al. Toward a low-cost, in-home, telemedicine-enabled assessment of disability in multiple sclerosis. *Mult Scler J*. 2019;25(11):1526-1534. doi:10.1177/1352458518793527

10. C. M, N. B, J. T, et al. Psychological impact of a remote psychometric consultation with hospitalized elderly people. *J Telemed Telecare*. 1997;3(3 PG-140-145):140-145. https://journals.sagepub.com/doi/pdf/10.1258/1357633971931048 NS  -.

11. C. MC, L.S. H, M. G, M. P, M.F. W. Teleneuropsychology: evidence for video teleconference-based neuropsychological assessment. *J Int Neuropsychol Soc*. 2014;20(10 PG-1028-1033):1028-1033. doi:http://dx.doi.org/10.1017/S1355617714000873

12. C. T, K. A, G. Z, et al. Virtual research visits in individuals with Parkinson disease enrolled in a clinical trial: React-PD study. *Neurology*. 2018;90(15 Supplement 1 PG-). NS  -.

13. C.J. B, N. S, P.M. M, J.P. W. Preliminary evaluation of a low-cost VideoConferencing (LCVC) system for remote cognitive testing of adult psychiatric patients. *Br J Clin Psychol*. 1993;32(3 PG-303-307):303-307. https://onlinelibrary.wiley.com/doi/abs/10.1111/j.2044-8260.1993.tb01060.x?sid=nlm%3Apubmed NS  -.

14. Carotenuto A, Rea R, Traini E, Ricci G, Fasanaro AM, Amenta F. Cognitive Assessment of Patients With Alzheimer’s Disease by Telemedicine: Pilot Study. *JMIR Ment Heal*. 2018;5(2 PG-e31):e31. doi:https://dx.doi.org/10.2196/mental.8097

15. Cullum CM, Weiner MF, Gehrmann HR, Hynan LS. Feasibility of telecognitive assessment in dementia. *Assessment*. 2006;13(4):385-390. doi:10.1177/1073191106289065

16. D.W. G, D.F. L, M. L, S. M, J.P. M. Skype: A tool for functional assessment in orthopaedic research. *J Telemed Telecare*. 2012;18(2 PG-94-98):94-98. doi:http://dx.doi.org/10.1258/jtt.2011.110814

17. Davis LE, Coleman J, Harnar J, King MK. Teleneurology: Successful Delivery of Chronic Neurologic Care to 354 Patients Living Remotely in a Rural State. *Telemed e-Health*. 2014;20(5):473-477. doi:10.1089/tmj.2013.0217

18. Davis LE, Harnar J, Lachey-Barbee LA, Pirio Richardson S, Fraser A, King MK. Using Teleneurology to Deliver Chronic Neurologic Care to Rural Veterans: Analysis of the First 1,100 Patient Visits. *Telemed e-Health*. 2019;25(4):274-278. doi:10.1089/tmj.2018.0067

19. DeYoung N, Shenal B V. The reliability of the Montreal Cognitive Assessment using telehealth in a rural setting with veterans. *J Telemed Telecare*. 2019;25(4 PG-197-203):197-203. doi:https://dx.doi.org/10.1177/1357633X17752030

20. E.R. D, J.D. W, M.T. B, et al. Feasibility of virtual research visits in fox trial finder. *J Parkinsons Dis*. 2015;5(3 PG-505-515):505-515. doi:http://dx.doi.org/10.3233/JPD-150549

21. F. C, P. B, M. T, H. M, H. C, R. D. Interrater agreement between telerehabilitation and face-to-face clinical outcome measurements for total knee arthroplasty. *Telemed J E Health*. 2010;16(3 PG-293-298):293-298. doi:http://dx.doi.org/10.1089/tmj.2009.0106

22. F. T, F. P, L. B, et al. Videoconference-based mini mental state examination: a validation study. *Telemed J E Health*. 2013;19(12 PG-931-937):931-937. doi:http://dx.doi.org/10.1089/tmj.2013.0035

23. Fraint A, Stebbins GT, Pal G, Comella CL. Reliability, feasibility and satisfaction of telemedicine evaluations for Cervical Dystonia. *J Telemed Telecare*. June 2019:1357633X19853140. doi:10.1177/1357633X19853140

24. Galusha-Glasscock JM, Horton DK, Weiner MF, Cullum CM. Video Teleconference Administration of the Repeatable Battery for the Assessment of Neuropsychological Status. *Arch Clin Neuropsychol*. 2016;31(1):8-11. doi:10.1093/arclin/acv058

25. Goldstein Y, Schermann H, Dolkart O, et al. Video examination via the smartphone: A reliable tool for shoulder function assessment using the constant score. *J Orthop Sci*. 2019;24(5 PG-812-816):812-816. doi:https://dx.doi.org/10.1016/j.jos.2018.12.023

26. Grosch MC, Weiner MF, Hynan LS, Shore J, Cullum CM. Video teleconference-based neurocognitive screening in geropsychiatry. *Psychiatry Res*. 2015;225(3):734-735. doi:10.1016/j.psychres.2014.12.040

27. H.-Y. P, S.-S. J, J.-Y. L, A.-R. C, J.H. P. Korean Version of the Mini-Mental State Examination Using Smartphone: A Validation Study. *Telemed J E Health*. 2017;23(10 PG-815-821):815-821. doi:http://dx.doi.org/10.1089/tmj.2016.0281

28. H. L, S. M, L. S, T.G. R. Validity and reliability of the assessment and diagnosis of musculoskeletal elbow disorders using telerehabilitation. *J Telemed Telecare*. 2012;18(7 PG-413-418):413-418. doi:http://dx.doi.org/10.1258/jtt.2012.120501

29. H.E. W, J.M. G-G, K.B. W, et al. Remote Neuropsychological Assessment in Rural American Indians with and without Cognitive Impairment. *Arch Clin Neuropsychol*. 2016;31(5 PG-420-425):420-425. doi:http://dx.doi.org/10.1093/arclin/acw030

30. Hildebrand R, Chow H, Williams C, Nelson M, Wass P. Feasibility of neuropsychological testing of older adults via videoconference: Implications for assessing the capacity for independent living. *J Telemed Telecare*. 2004;10(3):130-134. doi:10.1258/135763304323070751

31. Hubble JP, Pahwa R, Michalek DK, Thomas C, Koller WC. Interactive video conferencing: a means of providing interim care to Parkinson’s disease patients. *Mov Disord*. 1993;8(3 PG-380-2):380-382. NS  -.

32. I.V. V, B. N, A. C, et al. Telepsychiatry for neurocognitive testing in older rural latino adults. *Am J Geriatr Psychiatry*. 2015;23(7 PG-666-670):666-670. doi:http://dx.doi.org/10.1016/j.jagp.2014.08.006

33. J. W, M. W, J. F. Can a low-cost webcam be used for a remote neurological exam? *Stud Health Technol Inform*. 2013;190((Wallin) Georgetown University, Washington, DC, United States PG-30-32):30-32. doi:http://dx.doi.org/10.3233/978-1-61499-276-9-30

34. J.R. D, G.W. W, A.E. A. Telemedicine and the diagnosis of speech and language disorders. *Mayo Clin Proc*. 1997;72(12 PG-1116-1122):1116-1122. doi:http://dx.doi.org/10.4065/72.12.1116

35. K. J, M.C. H. Telehealth Videoconferencing for Children With Hemophilia and Their Families: A Clinical Project. *J Pediatr Oncol Nurs*. 2016;33(4 PG-282-288):282-288. doi:http://dx.doi.org/10.1177/1043454215607340

36. K.E. E, D.S. W, J.A. D, W. L, L. C, L.L. H. Diagnosis, diagnostic confidence, and management concordance in live-interactive and store-and-forward teledermatology compared to in-person examination. *Telemed e-Health*. 2008;14(9 PG-889-895):889-895. doi:http://dx.doi.org/10.1089/tmj.2008.0001

37. K.L. A, M.T. B, P. A, et al. Determining the reliability of performing the modified unified Parkinson’s disease rating scale (UPDRS) remotely in a pilot virtual visit study in the home. *Mov Disord*. 2015;30(SUPPL. 1 PG-S414):S414. doi:http://dx.doi.org/10.1002/mds.26295

38. Kirkwood KT, Peck DF, Bennie L. The consistency of neuropsychological assessments performed via telecommunication and face to face. *J Telemed Telecare*. 2000;6(3):147-151. doi:10.1258/1357633001935239

39. L. S, A. H, P. M, A. A, E. L, L. C. Reliability of telemedicine in the assessment of seriously ill children. *Pediatrics*. 2016;137(3 PG-e20150712):e20150712. doi:http://dx.doi.org/10.1542/peds.2015-0712

40. L. S, H. L, S. M, T.G. R. Assessment and diagnosis of musculoskeletal shoulder disorders over the internet. *Int J Telemed Appl*. 2012;((Steele, Lade, McKenzie, Russell) Division of Physiotherapy, School of Health and Rehabilitation Sciences, University of Queensland, Brisbane, QLD 4072, Australia PG-945745):945745. doi:http://dx.doi.org/10.1155/2012/945745

41. L. W, M. M-K, J. R, P. V, L.C. G. The Rowland universal dementia assessment scale (RUDAS) as a reliable screening tool for dementia when administered via videoconferencing in elderly post-acute hospital patients. *J Telemed Telecare*. 2012;18(3 PG-176-179):176-179. doi:http://dx.doi.org/10.1258/jtt.2012.SFT113

42. L.K. A, S. S, G. A, et al. Accuracy of remote video cellphone evaluation of stroke deficits using california brief stroke scale. *Stroke*. 2013;44(2 MeetingAbstract PG-). NS  -.

43. L.M. S, J. W, T. G, D. C, C. A, S.E. S. Physical Examinations via Video for Patients With Heart Failure: Qualitative Study Using Conversation Analysis. *J Med Internet Res*. 2020;22(2 PG-e16694):e16694. doi:http://dx.doi.org/10.2196/16694

44. Lesher J, Davis LS, Gourdin FW, Englis D, Thompson WO. Telemedicine evaluation of cutaneous diseases: A blinded comparative study. *J Am Acad Dermatol*. 1998;38(1):27-31. doi:10.1016/S0190-9622(98)70534-1

45. M. A, P.G. VH, A. A, et al. Telemedicine Physical Examination Utilizing a Consumer Device Demonstrates Poor Concordance with In-Person Physical Examination in Emergency Department Patients with Sore Throat: A Prospective Blinded Study. *Telemed J E Health*. 2018;24(10 PG-790-796):790-796. doi:http://dx.doi.org/10.1089/tmj.2017.0240

46. M. B, K. D, V. V, J. W, E.R. D, K. B. Conducting the montreal cognitive assessment remotely in Huntington’s disease. *Neurotherapeutics*. 2014;11(1 PG-224):224. doi:http://dx.doi.org/10.1007/s13311-013-0232-3

47. M. O, Y. O, T. U, et al. How Accurate Are First Visit Diagnoses Using Synchronous Video Visits with Physicians? *Telemed J E Health*. 2017;23(2 PG-119-129):119-129. doi:http://dx.doi.org/10.1089/tmj.2015.0245

48. M.F. W, H.C. R, K. H. Videoconference diagnosis and management of Choctaw Indian dementia patients. *Alzheimer’s Dement*. 2011;7(6 PG-562-566):562-566. doi:http://dx.doi.org/10.1016/j.jalz.2011.02.006

49. M.M. S, P. R-D, R.M. W, et al. Identifying undiagnosed dementia in residential care veterans: Comparing telemedicine to in-person clinical examination. *Int J Geriatr Psychiatry*. 2004;19(2 PG-101-108):101-108. doi:http://dx.doi.org/10.1002/gps.1029

50. M.T. B, R. D, K.M. B. Monitoring fluctuations in motor function and mood hourly in Parkinson’s disease via telemedicine. *Mov Disord*. 2014;29(SUPPL. 1 PG-S156):S156. doi:http://dx.doi.org/10.1002/mds.25914

51. Menon AS, Kondapavalru P, Krishna P, et al. Evaluation of a portable low cost videophone system in the assessment of depressive symptoms and cognitive function in elderly medically III veterans. *J Nerv Ment Dis*. 2001;189(6):399-401. doi:10.1097/00005053-200106000-00009

52. N.S. C, J.A. A, B.M. B, J.W. W, A.E. H. Assessing exercise capacity using telehealth: A feasibility study in adults with cystic fibrosis. *Respir Care*. 2013;58(2 PG-286-290):286-290. doi:http://dx.doi.org/10.4187/respcare.01922

53. Nachum S, Stern ME, Greenwald PW, Sharma R. Use of Physician-Guided Patient Self-Examination to Diagnose Appendicitis: A Telemedicine Case Report. *Telemed J E Health*. 2019;25(8 PG-769-771):769-771. doi:https://dx.doi.org/10.1089/tmj.2018.0115

54. P. G, A. V, I. R, et al. Two-year experience with telemedicine in the follow-up of patients in home peritoneal dialysis. *J Telemed Telecare*. 2007;13(6 PG-288-292):288-292. doi:http://dx.doi.org/10.1258/135763307781644906

55. P.K. L, M. D, L. F, S. M, P. G. Development of a telemedicine protocol for the diagnosis of Alzheimer’s disease. *J Telemed Telecare*. 2007;13(2 PG-90-94):90-94. doi:http://dx.doi.org/10.1258/135763307780096159

56. P.K. L, P. R, S. M, J. S, L. F, P. G. Can patients with dementia be assessed at a distance? The use of Telehealth and standardised assessments. *Intern Med J*. 2004;34(5 PG-239-242):239-242. doi:http://dx.doi.org/10.1111/j.1444-0903.2004.00531.x

57. R. M, C. L, G. B, R. M, W.-L. L, M. A. Comparing High Definition Live Interactive and Store-and-Forward Consultations to In-Person Examinations. *Telemed J E Health*. 2017;23(3 PG-213-218):213-218. doi:http://dx.doi.org/10.1089/tmj.2016.0093

58. R.C. B, P.H. Y, M.E. C, et al. Telemedicine versus face-to-face evaluations by respiratory therapists of mechanically ventilated neonates and children: A pilot study. *Respir Care*. 2016;61(2 PG-149-154):149-154. doi:http://dx.doi.org/10.4187/respcare.04080

59. R.E. K, A.W. S, M. K, et al. Virtual visits for Parkinson disease: A multicenter noncontrolled cohort. *Neurol Clin Pract*. 2017;7(4 PG-283-295):283-295. doi:http://dx.doi.org/10.1212/CPJ.0000000000000371

60. R.F. D, J.E. S. Virtual visits in a general medicine practice: A pilot study. *Telemed e-Health*. 2008;14(6 PG-525-530):525-530. doi:http://dx.doi.org/10.1089/tmj.2007.0101

61. R.L. J, M. M. Assessing elderly patients with congestive heart failure via in-home interactive telecommunication. *J Gerontol Nurs*. 2001;27(1 PG-21-27):21-27. doi:http://dx.doi.org/10.3928/0098-9134-20010101-09

62. Russell TG, Hoffmann TC, Nelson M, Thompson L, Vincent A. Internet-based physical assessment of people with Parkinson disease is accurate and reliable: a pilot study. *J Rehabil Res Dev*. 2013;50(5 PG-643-50):643-650. NS  -.

63. S. R, S. L, H. M, J. S. Tablet computers address limitations of telestroke systems. *Neurology*. 2014;82(10 SUPPL. 1 PG-). NS  -.

64. S.E. P, S.J. D, L. S, M. G, A. H. Televideo assessment using Functional Reach Test and European Stroke Scale. *J Rehabil Res Dev*. 2007;44(5 PG-659-664):659-664. doi:http://dx.doi.org/10.1682/JRRD.2006.11.0144

65. Singh SP, Arya D, Peters T. Accuracy of telepsychiatric assessment of new routine outpatient referrals. *BMC Psychiatry*. 2007;7. doi:10.1186/1471-244X-7-55

66. T. S, J. L, L. G, R. L, P. S. Could everyday technology improve access to assessments? A pilot study on the feasibility of screening cognition in people with Parkinson’s disease using the Montreal Cognitive Assessment via Internet videoconferencing. *Aust Occup Ther J*. 2016;63(6 PG-373-380):373-380. doi:http://dx.doi.org/10.1111/1440-1630.12288

67. T. S, J. L, L. G, R. L, P. S. Remotely assessing symptoms of Parkinson’s disease using videoconferencing: A feasibility study. *Neurol Res Int*. 2016;2016((Liddle, Lamont, Silburn) Asia-Pacific Centre for Neuromodulation, Queensland Brain Institute, UQ Centre for Clinical Research, University of Queensland, Brisbane, QLD, Australia PG-4802570):4802570. doi:http://dx.doi.org/10.1155/2016/4802570

68. T.C. C, L. A, P.S. M, et al. Assessing Cognitive Function in Older Adults Using a Videoconference Approach. *EBioMedicine*. 2016;11((Mariz) Emergency Department, Intermediate Care Unit (EDIMCU), Hospital de Braga, Braga, Portugal PG-278-284):278-284. doi:http://dx.doi.org/10.1016/j.ebiom.2016.08.001

69. Turner A. Case Studies in Physical Therapy: Transitioning A “Hands-On” Approach into A Virtual Platform. *Int J telerehabilitation*. 2018;10(1 PG-37-50):37-50. doi:https://dx.doi.org/10.5195/ijt.2018.6253

70. V. V, S.J. D, K.M. B, P. W, E.R. D. Virtual visits for Parkinson disease: A case series. *Neurol Clin Pract*. 2014;4(2 PG-146-152):146-152. doi:http://dx.doi.org/10.1212/01.CPJ.0000437937.63347.5a

71. Van Hooff RJ, Cambron M, Van Dyck R, et al. Prehospital unassisted assessment of stroke severity using telemedicine: A feasibility study. *Stroke*. 2013;44(10):2907-2909. doi:10.1161/STROKEAHA.113.002079

72. Van Hooff RJ, De Smedt A, De Raedt S, et al. Unassisted assessment of stroke severity using telemedicine. *Stroke*. 2013;44(5):1249-1255. doi:10.1161/STROKEAHA.111.680868

73. Vestal L, Smith-Olinde L, Hicks G, Hutton T, Hart J. Efficacy of language assessment in Alzheimer’s disease: comparing in-person examination and telemedicine. *Clin Interv Aging*. 2006;1(4):467-471. doi:10.2147/ciia.2006.1.4.467

74. W. M, A. K, D.G. M, M. C, C. H. Reliability of the MMSE administered in-person and by telehealth. *Can J Neurol Sci*. 2008;35(5 PG-643-646):643-646. doi:http://dx.doi.org/10.1017/S0317167100009458
